# Prioritizing genes associated with brain disorders by leveraging enhancer-promoter interactions in diverse neural cells and tissues

**DOI:** 10.1186/s13073-023-01210-6

**Published:** 2023-07-24

**Authors:** Xingzhong Zhao, Liting Song, Anyi Yang, Zichao Zhang, Jinglong Zhang, Yucheng T. Yang, Xing-Ming Zhao

**Affiliations:** 1 Institute of Science and Technology for Brain-Inspired Intelligence, and Department of Neurology of Zhongshan Hospital, Fudan University, 220 Handan Road, Shanghai, 200433 China; 2 MOE Key Laboratory of Computational Neuroscience and Brain-Inspired Intelligence, and MOE Frontiers Center for Brain Science, Fudan University, Shanghai, 200433 China; 3 State Key Laboratory of Medical Neurobiology, Institutes of Brain Science, Fudan University, Shanghai, 200032 China; 4 Internatioal Human Phenome Institutes (Shanghai), Shanghai, 200433 China

**Keywords:** *Cis*-regulatory element, Enhancer-promoter interaction, Bain disorder, Heritability

## Abstract

**Background:**

Prioritizing genes that underlie complex brain disorders poses a considerable challenge. Despite previous studies have found that they shared symptoms and heterogeneity, it remained difficult to systematically identify the risk genes associated with them.

**Methods:**

By using the CAGE (Cap Analysis of Gene Expression) read alignment files for 439 human cell and tissue types (including primary cells, tissues and cell lines) from FANTOM5 project, we predicted enhancer-promoter interactions (EPIs) of 439 cell and tissue types in human, and examined their reliability. Then we evaluated the genetic heritability of 17 diverse brain disorders and behavioral-cognitive phenotypes in each neural cell type, brain region, and developmental stage. Furthermore, we prioritized genes associated with brain disorders and phenotypes by leveraging the EPIs in each neural cell and tissue type, and analyzed their pleiotropy and functionality for different categories of disorders and phenotypes. Finally, we characterized the spatiotemporal expression dynamics of these associated genes in cells and tissues.

**Results:**

We found that identified EPIs showed activity specificity and network aggregation in cell and tissue types, and enriched TF binding in neural cells played key roles in synaptic plasticity and nerve cell development, i.e., EGR1 and SOX family. We also discovered that most neurological disorders exhibit heritability enrichment in neural stem cells and astrocytes, while psychiatric disorders and behavioral-cognitive phenotypes exhibit enrichment in neurons. Furthermore, our identified genes recapitulated well-known risk genes, which exhibited widespread pleiotropy between psychiatric disorders and behavioral-cognitive phenotypes (i.e., FOXP2), and indicated expression specificity in neural cell types, brain regions, and developmental stages associated with disorders and phenotypes. Importantly, we showed the potential associations of brain disorders with brain regions and developmental stages that have not been well studied.

**Conclusions:**

Overall, our study characterized the gene-enhancer regulatory networks and genetic mechanisms in the human neural cells and tissues, and illustrated the value of reanalysis of publicly available genomic datasets.

**Supplementary Information:**

The online version contains supplementary material available at 10.1186/s13073-023-01210-6.

## Background

Cell-type-specific variations in the epigenetic regulation of gene expression are critical to the development and maintenance of a healthy human brain. It has been shown that, instead of protein-coding sequence, GWAS variants are enriched in the *cis*-regulatory elements (CREs; e.g., enhancers and promoters) of cell types that are relevant to the pathophysiological basis of a given trait [1]. The risk genes are assumed to have cell-type-specific effects for both neural and non-neural cell types [2, 3]. In addition, recent single-cell studies indicated transcriptomic and epigenomic changes at cell-type-specific level in several brain disorders [4–7]. Thus, studying the regulatory networks of CREs (i.e., enhancer-promoter interactions (EPIs)) at the cell-type-specific level is an important means to further understand both fundamental brain biology and the genetic basis of brain disorders and behavioral-cognitive phenotypes.

Despite its importance, the association between CREs as well as EPIs in human brain and their dysregulation in brain disorders are still poorly understood. Even when available, most previous studies have generally focused on a few cell types (e.g., neurons, astrocytes and microglia) or brain regions (e.g., dorsolateral prefrontal cortex and hippocampus) restricted in specific developmental stages (e.g., early fetal and adult period) [5, 8–14]. Most of these studies leveraged chromatin accessibility (DNase-seq and/or ATAC-seq) data and histone mark ChIP-seq data to define a collection of active CREs in human brain, followed by typically predicting the EPIs using statistical associations (i.e., activity correlation) [15–17] or more sophisticated computational approaches (e.g., machine learning-based methods) [18–21]. However, DNase-seq or ATAC-seq technologies can only detect open chromatin regions, a small fraction of which could function as active enhancers. Although ChIP-seq technologies can systematically profile histone modifications, it is still challenging to accurately distinguish active enhancers from poised ones from the histone modification profiles. By contrast, the CAGE (Cap Analysis of Gene Expression) technology can accurately detect the 5′ ends of capped RNAs, which represent transcriptional activity of genes. More importantly, the active enhancer regions can also generate capped RNAs (i.e., enhancer RNAs) [22, 23], which can be also detected by CAGE technology. This property has been successfully used in identifying active enhancers in different cell and tissue types [24, 25].

FANTOM5 project has generated CAGE profiles from hundreds of diverse human samples, including organs, primary cell types, and cancer cell lines [26]. Among them, we noticed that a wealth of samples from human brain have been profiled, covering major neural cell types and brain regions from different developmental stages. These datasets make it possible to systematically quantify the activity of CREs and reconstruct EPIs in human brain. However, there are currently limited efforts to leverage the rich data in human brain from FANTOM5 project to analyze the activity specificity of CREs and network architecture of EPIs in human brain, and further elucidate the risk genes of brain disorders.

**Fig. 1:**
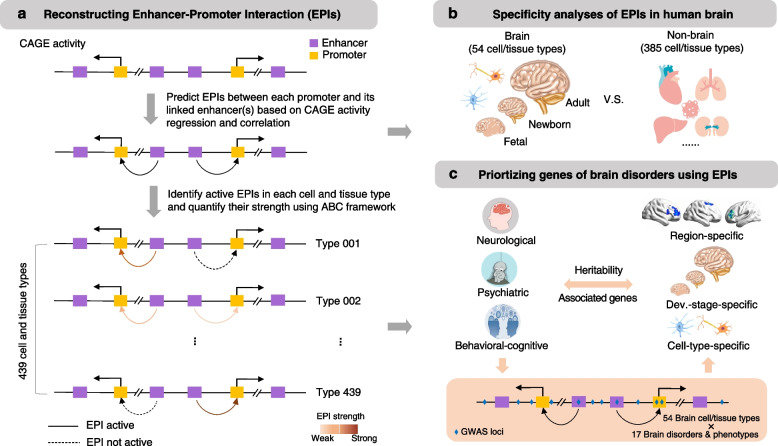
Outline of the study. **a** Schematic showing the method for reconstructing EPIs in each cell and tissue type. In total, we included 958 human samples from FANTOM5 project (Table S1), which can be further grouped into 439 different cell and tissue types (Table S2). **b** Analysis of the reconstructed EPIs showing their tissue specificity (i.e., comparing between brain and non-brain samples) and developmental stage (i.e., comparing between fetal, newborn and adult samples) specificity. **c** The EPIs reconstructed in the neural cell and tissue types were used to prioritize the associated genes of 17 different neurological disorders, psychiatric disorders, and behavioral-cognitive phenotypes by integrating with their GWAS summary statistics data

To explore these ideas, here we first reconstruct active interactions between enhancers and their target promoters in 439 different cell and tissue types from the FANTOM5 project [27] (Fig. 1a). We then systematically analyze the tissue and regional specificity of the activity and their networks of enhancers and promoters from neural cell and tissue types (Fig. 1b). Furthermore, we prioritize a set of critical genes associated with brain disorders and behavioral-cognitive phenotypes in a cell- and region-specific manner, expanding our understanding on the functionality of some brain regions that have not been well studied (Fig. 1c). Our analyses reveal how the unbiased data-driven analysis of publicly available data can be used to infer the EPIs in human brain and nominate the genes with substantial risk potential of brain disorders in diverse neural cell types and brain regions in different developmental stages.

## Methods

### Collection of CAGE datasets and categorization of samples

We downloaded the CAGE read alignment files for 958 human cell and tissue types (including primary cells, tissues, and cell lines) from FANTOM5 project (see https://fantom.gsc.riken.jp/5/datafiles/reprocessed/hg38_v9/basic) [27]. The annotations of these samples (e.g., biosample type, age, life stage and sex) were also obtained from FANTOM5 project (Additional file 2: Table S1). All the 958 samples were annotated as 439 different cell and tissue types (i.e., states) by aggregating replicates, and then categorized into 34 cell and tissue groups (Additional file 2: Table S1). Among all these samples, there are 93 samples covering 54 different cell and tissue types from brain.

All genomic coordinates in this study were based on the human reference genome hg38. The genomic coordinates that were originally provided in hg19 by their publications or resource were converted to hg38 using the liftover tool of the UCSC Genome Browser [28].

### Data processing and reconstruction of EPIs

For each sample from FANTOM5 project, we computed the CAGE TPM value (reads coverage per million mapped reads) at the flanking regions (from 500 bp upstream to 500 bp downstream) of the TSS of each protein-coding gene from GENCODEv38 [29]. We then removed the genes located in ChrY and ChrM. Finally, we obtained a list of 19,878 promoters. In each sample, we then determined a set of promoters that are active and in usage by thresholding the TPM value larger than 1. We also downloaded the processed CAGE-derived enhancers and their activity signal in each sample from FANTOM5 website (see https://fantom.gsc.riken.jp/5/datafiles/reprocessed/hg38_v9/extra/enhancer/). In total, we obtained a list of 63,285 enhancers. The usage information of the enhancers in each sample is also obtained from FANTOM5 website. As expected, the enhancers and promoters exhibit strong cell and tissue specificity (Gini index > 0.8) (Additional file 1: Fig. S1).

We developed a simple and multi-step pipeline to predict and quantify the EPIs in each sample and in each cell and tissue type. Here we briefly describe the procedures as follows:

- (1) We applied a LASSO regression model that could predict its activity signal by using its all candidate regulating enhancers within 1 Mb of it, as in Cao et al. [21]. Based on the results of the LASSO regression model, the subset of enhancers of which the CAGE activity positively correlates with the CAGE activity of putative target promoters (i.e., based on the β coefficients in the LASSO regression) were retained, indicating that these enhancers might have potential regulating association with their target promoter.
- (2) We filtered the candidate enhancers for each promoter based on their pairwise activity correlation (Spearman correlation coefficient), asking that the observed activity correlation should be higher than the random enhancer-promoter pairs at an empirical false discovery rate (FDR) of 5%. For each promoter, the random enhancer-promoter pairs were constructed by randomly selecting 10,000 enhancers located in all the other chromosomes where the promoter was from. This step can further minimize the false positive in the candidate enhancers from the first step (Additional file 1: Fig. S2).
- (3) We then reconstructed EPIs for each sample based on the enhancer and promoter usage in the sample. For each candidate EPI retained from the last step, it could be defined as active and inactive in the sample based on whether both of its enhancer and promoter were determined as in usage in that sample.
- (4) For each sample, we applied the Activity-By-Contact (ABC) model [30] to quantify the EPIs reconstructed in the third step. Briefly, we computed the ABC scores for each EPI by combining the enhancer activity, the promoter activity, and the distance between the enhancer and promoter, as well as the background ABC scores for the promoter (Additional file 1: Fig. S3).
- (5) The active EPIs from the replicate samples were aggregated to define a set of active EPIs for each cell and tissue type. For the EPIs that were determined as active in more than one replicate sample, we assigned the maximal ABC score to the corresponding EPI when aggregating for each cell and tissue type.

### Validation of CAGE-derived enhancers as active enhancers

We evaluated the enrichment of the CAGE-derived enhancers with the active and poised enhancers defined using histone mark ChIP-seq and ATAC-seq/scATAC-seq technologies. Notably, we used both brain-specific datasets and whole-body datasets to estimate the enrichment in the brain samples and all samples from FANTOM5 project, respectively.

For the datasets generated by histone mark ChIP-seq technologies, the whole-body datasets were obtained from EpiMap (see https://personal.broadinstitute.org/cboix/epimap/ChromHMM/observed_aux_18_hg38/CALLS/) [31], among which the brain samples were used in the brain-specific analysis. In the datasets from EpiMap [31], the annotations EnhG1 (state_7), EnhG2 (state_8), EnhA1 (state_9), EnhA2 (state_10), EnhWk (state_11), and EnhBiv (state_15) were regarded as the background, while the two annotations among them, EnhA1 (state_9) and EnhA2 (state_10) were regarded as active enhancers.

We intersected the genomic coordinates of the CAGE-derived enhancers from FANTOM5 project with the enhancer regions defined by histone mark ChIP-seq, and then estimated the enrichment of CAGE-derived enhancers from FANTOM5 project and the active enhancers using hypergeometric test. We also calculated an OR score as.

$$OR=\frac{{CAGE}_{act}/{CAGE}_{bgd}}{{ChIP}_{act}/{ChIP}_{bgd}}$$

where *CAGE*_*act*_ is the number of active enhancers in a specific cell/tissue type from FANTOM5 project, *CAGE*_*bgd*_ is the number of total enhancers from FANTOM5 project, *ChIP*_*act*_ is the number of active enhancers from EpiMap, and *ChIP*_*bgd*_ is the number of total enhancers from EpiMap.

For the datasets generated by ATAC-seq/scATAC-seq technologies, the whole-body datasets were obtained from CATlas (see http://yed.ucsd.edu:8787) [32], while the brain-specific datasets were obtained from Markenscoff-Papadimitriou et al. [33]. In the study by Markenscoff-Papadimitriou et al. [33], the open chromatin regions were regarded as the background, while the predicted regulatory elements were regarded as active enhancers. In the datasets from CATlas [32], the total cCREs were regarded as the background, while the gene-linked cCREs were regarded as active enhancers in human body. We intersected the genomic coordinates of the CAGE-derived enhancers from FANTOM5 project with the cCREs or open chromatin regions defined by ATAC-seq/scATAC-seq, and then estimated the enrichment of CAGE-derived enhancers from FANTOM5 project and the active enhancers using hypergeometric test. We also calculated an odds ratio (OR) score as.

$$OR=\frac{{CAGE}_{act}/{CAGE}_{bgd}}{{ATAC}_{act}/{ATAC}_{bgd}}$$

where *CAGE*_*act*_ is the number of active enhancers in a specific cell/tissue type from FANTOM5 project, *CAGE*_*bgd*_ is the number of total enhancers from FANTOM5 project, *ATAC*_*act*_ is the number of active enhancers from ATAC-seq/scATAC-seq, and *ATAC*_*bgd*_ is the number of total cCREs or open chromatin regions from ATAC-seq/scATAC-seq.

### cis-QTL enrichment analysis

We estimated the *cis*-QTL (including *cis*-expression QTL and *cis*-splicing QTL) enrichment in the CREs by calculating an OR score using the numbers of real *cis*-QTL variants and control variants located in the CREs compared to those in the baseline regions as.

$$OR=\frac{\mathrm{a}/\mathrm{b}}{\mathrm{c}/\mathrm{d}}$$

where *a* is the number of *cis*-QTL variants in the CREs; *b* is the number of control variants in the CREs; *c* is the number of *cis*-QTL variants in the baseline region; and *d* is the number of control variants in the baseline region.

The *cis*-QTL variants were downloaded from GTEx v8 (see https://gtexportal.org/home/datasets#filesetFilesDiv14) [34]. The baseline regions are the union of all the functional and putative functional regions in the human genome, including coding regions, untranslated regions, noncoding RNA genes, open chromatin regions, TF binding sites, active and repressed histone peaks from multiple tissue and cell types, and evolutionary conserved regions [35]. The set of control variants was generated with the same number and same MAF distribution as the real QTL variants, and this procedure was repeated 30 times to calculate a standard deviation for the variant enrichment.

To minimize the bias effects from LD structure, we also estimated the enrichment using the fine-mapped *cis*-eQTLs from GTEx v8 and observed a higher enrichment in the case of fine-mapped *cis*-eQTLs. We obtained fine-mapped *cis*-eQTLs with a posterior probability > 0.8 from the three GTEx fine-mapping *cis*-eQTL catalogs (CAVIAR, CaVEMaN, and DAP-G; see https://gtexportal.org/home/datasets#filesetFilesDiv15). Then, we applied the enrichment analysis pipeline as we did in the case of total *cis*-eQTLs on these fine-mapped *cis*-eQTLs.

### Evaluation of the reconstructed EPIs

We assessed the reconstructed EPIs using two independent datasets, i.e., GTEx *cis*-eQTLs and pcHi-C data from the matched and related cell and tissue types (Additional file 2: Table S3). For the *cis*-eQTLs, we obtained the datasets from GTEx V8 (see https://gtexportal.org/home/datasets#filesetFilesDiv14) [34]. We first intersected the genomic coordinates of the *cis*-eQTLs with the enhancer regions, and therefore the enhancers can be associated to their target promoters based on the target genes of *cis*-eQTLs provided by GTEx. Notably, for some tissue types in GTEx, their *cis*-eQTLs were inferred using very limited number of samples, which could result in significant incompleteness for the predicted *cis*-eQTLs. Thus, we only included the tissue types with sample size larger than 500 in this analysis. For the pcHi-C datasets, we first downloaded the significant P-O interactions (i.e., interactions between promoters and “other regions”) from GSE86189 [36]. In the pcHi-C dataset, the majority of the “other regions” linked to promoters are assumed to be putative enhancer regions. We then intersected the genomic coordinates of the “other regions” with the enhancer regions from FANTOM5 project to associate the enhancers to their target promoters.

For each FANTOM5 cell/tissue type for which *cis*-eQTLs or pcHi-C data were available, we then computed the area under the precision-recall (AUPR) of our predicted EPIs using the two independent validation sets as true labels. Notably, only the enhancers within 1 Mb from the promoters and that were available in the validation sets for at least once were considered in this analysis. As comparison, we also computed the AUPRs for another three alternative approaches: (1) linking enhancers to the closest promoter; (2) linking enhancers to the promoter with the maximum activity correlation (Spearman correlation coefficient); (3) randomly linking enhancers to the promoter within 1 Mb from it.

### Hierarchical clustering of EPIs among cell and tissue types

We evaluated the similarity of the EPIs between the cell and tissue types by calculating the extended Jaccard index [37] of the two sets of edges (i.e., EPIs) from the two cell and tissue types as.

$$f\left(a,b\right)=\frac{ab}{{|a|}^{2}+{|b|}^{2}-ab}$$

where* a* and* b* are the ABC-score-defined edge-weight vectors of the two sets of edges. We then used the distance matrix (i.e., 1 − *f*) to perform hierarchical clustering for all the cell and tissue types.

### Estimation of expression specificity of genes

We estimated the expression specificity score of each gene by calculating the Shannon entropy based on the activity values of the promoter across all the samples, as in Cao et al. [21]. For a promoter *i* with expression level *y*_*ij*_ in sample *j*, the overall expression specificity score was calculated as.

$$S\left(i\right)={\mathrm{log}}_{2}m+\sum_{j=1}^{m}[\frac{{y}_{ij}}{\sum_{l=1}^{m}{y}_{il}}{\mathrm{log}}_{2}\frac{{y}_{ij}}{\sum_{l=1}^{m}{y}_{il}}]$$

where *m* is the total number of samples.

### Identification of CAGE activity elevated and specific CREs in human brain

We applied DESeq2 [38] to detect enhancers and promoters with elevated activity in brain versus other tissues, and in a specific developmental stage versus the other developmental stages. Notably, we performed this analysis based on the CAGE count number of the enhancers and promoters. The enhancers and promoters which have an FDR < 0.05 and a fold change > 2 were considered as elevated ones. The enhancers regulating genes, promoters regulated by enhancers, and EPIs that are identified as active in > 50% samples in the corresponding cell and tissue type and in < 50% samples in all other cell and tissue types were regarded as tissue-specific ones.

### Visualization of tissue-specific EPIs

We first aggregated the EPIs from individual cell and tissue types into a global network, consisting of enhancers (nodes), promoters (nodes), and interactions between them (edges). For better visualization, we then created a virtual node for each cell and tissue type, and connected the virtual nodes to all the real nodes (i.e., enhancers and promoters) that are specifically active in the corresponding cell and tissue type using virtual edges (not shown in figure). Finally, the networks were arranged with perfuse force-directed layout. In the figure, we only presented the enhancer-promoter networks on Chr1. We visualized the networks using Cytoscape [39].

### TF motif enrichment analysis

We used findMotifsGenome in HOMER [40] with default parameters to identify significantly (FDR < 0.05) enriched TF motif occurrence in the enhancer regions with specific activity patterns in human brain. Both previously known (JASPAR2020_CORE_vertebrates [41]) and de novo enriched TF motifs were considered in the prediction.

### GWAS summary datasets of brain disorders and behavioral-cognitive phenotypes

We collected a comprehensive set of GWAS summary statistics for 17 brain disorders and behavioral-cognitive phenotypes (Additional file 2: Table S7). Most of these GWAS were available recently and based on meta-analyses with large sample sizes; the average sample size is over 300,000 individuals.

Here we show the sample size for each of the GWAS summary datasets as follows:

- Neurological disorders. (1) Alzheimer’s disease (AD) [42]: 7428 cases and 429,961 controls; (2) amyotrophic lateral sclerosis (ALS) [43]: 12,577 cases and 23,475 controls; (3) epilepsy [44]: 15,212 cases and 29,677 controls; (4) multiple sclerosis (MS) [45]: 47,429 cases and 68,374 controls; (5) Parkinson’s disease (PD) [46]: 37,700 cases and 1,400,000 controls.
- Psychiatric disorders. (1) Attention deficit hyperactivity disorder (ADHD) [47]: 19,099 cases and 34,194 controls; (2) anxiety [48]: 7016 cases and 14,754 controls; (3) autism spectrum disorder (ASD) [49]: 18,381 cases and 27,969 controls; (4) alcohol use disorder (AUD) [50]: 5408 cases and 121,604 controls; (5) bipolar disorder (BIP) [51]: 20,352 cases and 31,538 controls; (6) obsessive–compulsive disorder (OCD) [52]: 2688 cases and 7037 controls; (7) schizophrenia (SCZ) [53]: 54,418 cases and 78,818 controls; (8) Tourette’s syndrome (TS) [54]: 4819 cases and 9488 controls.
- Behavioral-cognitive phenotypes. (1) Insomnia [55]: 386,533 individuals; (2) intelligence [56]: 269,867 individuals; (3) neuroticism [57]: 390,278 individuals; (4) risk behavior [58]: 975,353 individuals.

### Heritability enrichment in the CREs in human brain

We applied partitioned LDSC [35] to estimate the heritability enrichment in the CREs (i.e., enhancers and promoters) that are active in each brain cell and tissue type. Briefly, LDSC regresses GWAS *χ*^2^ statistics on SNPs’ LD scores, which can reflect the degree to which each SNP is correlated with its surrounding SNPs [35, 59]. The partitioned LDSC approach can estimate the relative enrichment of heritability in specific genomic regions compared to the genomic background. The pre-calculated genome-wide LD scores were obtained from LDSC (https://data.broadinstitute.org/alkesgroup/LDSCORE/). The LD scores were calculated based on data from individuals of European ancestry from the 1000 Genomes Project [60]. We also removed the SNPs that were not annotated in HapMap3 [61] and those located in the major histocompatibility complex regions.

### Predicting the genes associated with brain disorders and behavioral-cognitive phenotypes based on EPIs

We applied our post-GWAS method nMAGMA [62] to aggregate the genetic signal from the related SNPs to their target genes. The SNPs located in the gene body and flanking (from 2 kb upstream to 2 kb downstream) regions were directly assigned to the corresponding gene. The SNPs located in the distal enhancers were assigned to their target genes based on the reconstructed EPIs. We performed these analyses separately for each of the 55 cell and tissue types from human brain. Finally, we predicted a set of candidate significant genes (FDR < 0.05) associated with brain disorders and behavioral-cognitive phenotypes in each brain cell and tissue type.

The candidate associated genes were further filtered based on their expression specificity. Briefly, we first defined a set of relatively highly expressed genes for each sample requiring that their TPM > 1 and higher than 50% of the remaining genes in each sample. These relatively highly expressed genes from the replicate samples were aggregated to form a set of relatively highly expressed genes for each brain cell and tissue type. Finally, the associated genes for each brain cell and tissue type were defined by intersecting the candidate associated genes with these relatively highly expressed genes from the corresponding cell and tissue type.

### Collection of risk genes of brain disorders

The known risk genes for the brain disorders were collected from diverse resources as follows: (1) the risk genes of ADHD were collected from ADHDgene database (http://adhd.psych.ac.cn), followed by selecting those having support by at least 60% of all the studies included in the database [63]; (2) the risk genes of ASD were downloaded from AutDB database (http://autism.mindspec.org/autdb) and combined with the risk genes from recent studies [64, 65]; (3) the risk genes of SCZ were obtained from SZGene database (http://www.szgene.org/) and Wang et al. [6, 66]; (4) the risk genes of BIP were obtained from DisGeNet [67]; (5) the risk genes of MDD were downloaded from Polygenic pathways database (http://www.polygenicpathways.co.uk/depression.htm); (6) the risk genes of AD were obtained from ALzGene database (http://www.alzgene.org) [68]; (7) the risk genes of PD were downloaded from PDGene database (http://www.pdgene.org) [69]. The complete list of these known risk genes can be found in Additional file 2: Table S9.

### Expression analysis of the associated genes

We assessed the expression levels of the genes associated with the brain disorders and behavioral-cognitive phenotypes. We leveraged both bulk and single-cell transcriptomic data. For the single-cell data, we used the single-cell gene expression data in human brain from our STAB database [70]. Considering the sample number available in the database, we used datasets from two developmental time windows (i.e., 19–24 PCW, 40–60 years) and three cell types (i.e., Astro, Neuron, OPC). In addition, we obtained the bulk gene expression data during human brain development from Zhu et al. [71]. We defined a set of developmental time windows by merging the similar developmental periods as in Zhu et al. (Additional file 2: Table S12).

## Results

### Reconstruction of EPIs in diverse human cell and tissue types

Assigning enhancers to their target genes on a genome-wide scale remains a difficult task. Although quite a few computational methods have been developed for predicting EPIs [15–21], most of them need a wealth of functional genomics data such as DNase-seq data, ATAC-seq, and/or histone mark ChIP-seq data. Here, we aim to use only CAGE activity of enhancers and promoters from the FANTOM5 datasets [27] (Additional file 1: Fig. S1). Notably, the enhancer regions used in this study were defined by the CAGE data and obtained from ref. [24]. We confirmed that these CAGE-derived enhancers were significantly enriched with the active enhancers defined based on histone mark ChIP-seq (*P* < 2.2e − 16, hypergeometric test; Additional file 1: Fig. S2) and ATAC-seq (*P* < 2.2e − 16, hypergeometric test; Additional file 1: Fig. S3) datasets from human body. Many of the existing methods for predicting EPIs were built on sophisticated machine learning-based models, which cannot be simply applied on the hundreds of samples from the FANTOM5 project [27]. Therefore, we set up a deliberately simple pipeline for determining the target promoters of enhancers in each of the 958 FANTOM5 samples.

Our reconstruction pipeline was generally built based on previous studies showing that real EPIs presented significantly higher activity correlations between enhancers and their target promoters, and also, a gene can be regulated by multiple enhancers [15, 72]. Briefly, we designed the pipeline by integrating the advantages from both correlation- and regression-based approaches, which involves four main steps (Fig. 1a, Additional file 2: Table S1 and Methods). In the first step, it predicts the subset of enhancers of which the activity positively correlates with the activity of putative target promoters using multiple regression of all enhancers in the genomic neighborhood of a promoter [21]. In the second step, it computes the activity correlation for each potential EPI across all samples and discards the EPIs with relatively lower activity correlation (Additional file 1: Fig. S4). In the third step, it identifies active EPI for each sample by pruning the potential EPIs that are with inactive enhancers and/or promoters in a specific sample. In the fourth step, we used the Activity-By-Contact (ABC) framework [30] to quantify the interaction strength for each active EPI in each sample. Finally, the active EPIs from the replicate or similar samples were aggregated to define the set of active EPIs for each of the 439 different cell and tissue types (provided on our supplementary website; URL: https://soulnature.github.io/brainepl).

Overall, we identified 36,807 EPIs, involving 16,872 unique enhancers and 8077 unique promoters. Every reconstructed enhancer-promoter network has up to 11,610 active enhancers (on average 3292 enhancers), 6275 active promoters (on average 3937 promoters), and 24,921 EPIs (on average 9145 interactions) (Additional file 2: Table S2).

### Global architecture and reliability of the reconstructed EPIs

**Fig. 2:**
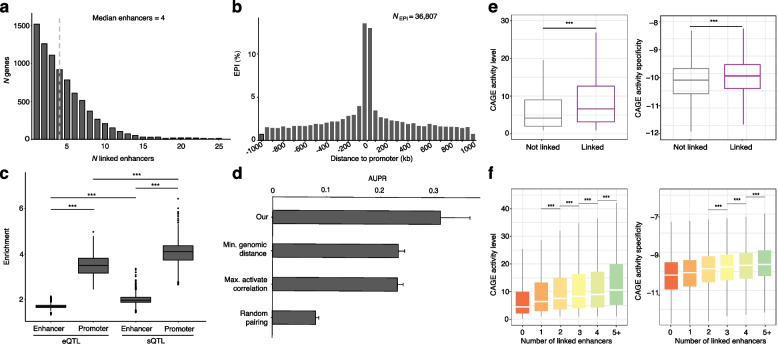
Reliability of the reconstructed EPIs. **a** Histogram showing the number of genes that were predicted as linked with a range number of enhancers. **b** Histogram showing the distribution of distance between the enhancers to their target promoters for the reconstructed EPIs. **c** Odds ratio enrichment of *cis*-eQTL and *cis*-sQTL variants [34] in the promoter-linked enhancers and enhancer-linked promoters, respectively. *** *P* < 0.001; Wilcoxon test. **d** The accuracy of different EPI prediction approaches. We estimated the accuracy of the reconstructed EPIs using Area Under the Precision-Recall (AUPR) based on the pcHi-C data from 24 matching and related cell and tissues from the FANTOM5 project (Table S3). **e** Boxplots showing the CAGE activity and specificity of the promoters linked or not linked to enhancers. **f** Boxplots showing the CAGE activity and specificity of the promoters linked to a range number of enhancers. *** *P* < 0.001; Wilcoxon test in **e** and **f**

Over 82% of all linked promoters were linked to multiple enhancers with a median of 4 of the number of linked enhancers, and a large proportion of promoters are linked to single enhancer (Fig. 2a). This finding is consistent with the data from promoter capture Hi-C (pcHi-C) experiments [73]. Of the promoter-linked enhancers, about 34% are linked to a flanking promoter (within a 100-kb window, Fig. 2b), which is consistent with previous analyses [74], highlighting the importance of chromatin structure in determining the regulatory targets of enhancers. Next step, we evaluated the degree to which the *cis*-expression QTL (*cis*-eQTL) or *cis*-splicing QTL (*cis*-sQTL) variants from GTEx [34] are enriched in the linked enhancers and promoters (Fig. 2c, Additional file 1: Fig. S5 and “Methods”). The enrichment was considerably stronger in the linked promoters than in the linked enhancers, especially, as expected, for *cis*-sQTL variants. We also confirmed that the active EPIs reconstructed in each cell and tissue type exhibited significantly higher interaction strength than the inactive EPIs in the corresponding cell and tissue type (Additional file 1: Fig. S6), suggesting that our pipeline could not only assign enhancers to their target genes but also quantify their interaction strength.

Having shown that the global architecture of the reconstructed EPIs are reliable, we next sought to examine their accuracy using the chromatin loops derived from the pcHi-C data in 24 matching and related cell and tissues [75] (Additional file 2: Table S3, Table S4 and “Methods”). Overall, about 27% of the reconstructed EPIs were confirmed by the pcHi-C data, showing an improvement on results obtained by alternative approaches such as assigning enhancers to the closest, most strongly correlated target promoter or the random pairs (Fig. 2d and “Methods”). Using the same approach as for pcHi-C data, we also assessed our reconstructed EPIs in 37 matching and related tissues for which *cis*-eQTL data were available from GTEx [34] (Additional file 1: Fig. S7, Additional file 2: Table S3, Table S4 and Methods). Overall, about one-fourth of the EPIs were supported by a *cis*-eQTL variant, which is slightly better than the results from alternative approaches. Collectively, these results indicated that the reconstructed EPIs could reliably represent the physical interactions between enhancers and their target promoters in the specific samples.

Next, we asked whether the activity level from the CAGE activity data could support the reliability of the reconstructed EPIs. We found that the promoters with linked enhancers exhibited significantly elevated activity than those without linked enhancers (*P* < 2.22e − 16, Wilcoxon test) (Fig. 2e). We further observed that the promoters with more linked enhancers showed stronger activity level than those with fewer linked enhancers (Fig. 2f), which is consistent with previous studies [21]. Likewise, the promoters with more linked enhancers exhibited elevated activity specificity than those with fewer linked enhancers (Fig. 2e, f), indicating the strong lineage specificity of enhancers in EPIs [37, 76–78].

### Lineage specificity of the reconstructed EPIs

**Fig. 3:**
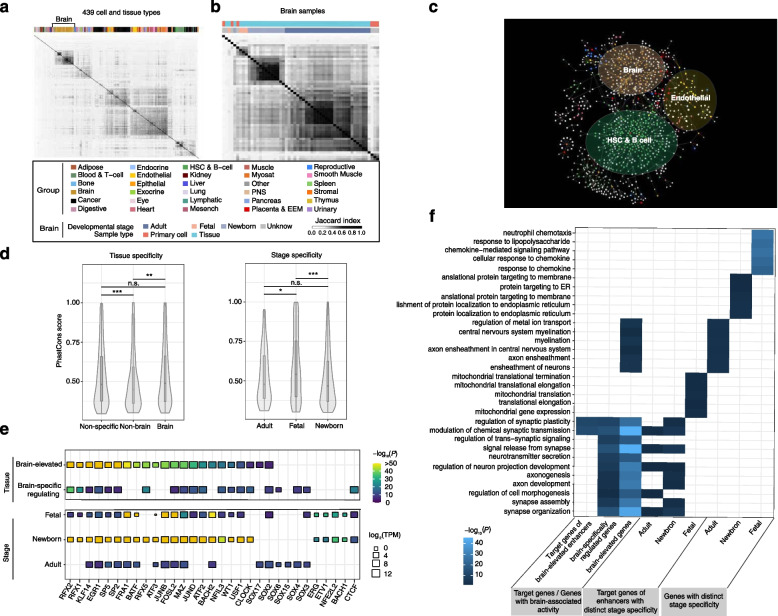
Specificity and functions of CREs and reconstructed EPIs in human brain. Clustering of the 439 cell and tissue types (**a**) and the 54 neural cell and tissue types (**b**) based on the similarities of their reconstructed EPIs. We used Jaccard index to quantify the overlap of the reconstructed EPIs bwteen each pair of cell and tissue types. **c** Visualization of the EPIs that were active in only one single group of cell and tissue types. Here we only presented the subnetworks on Chromosome1. **d** Evolutionary conservation of the enhancers with distinct tissue- (left) and stage-specific (right) activity patterns. *** *P* < 0.001; ** *P* < 0.01; * *P* < 0.05; Wilcoxon test. **e** The enrichment of TF binding in the enhancers with distinct tissue- (up) and stage-specific activity patterns (bottom). We showed the most enriched 15 TFs for each group of enhancers. **f** The most enriched biological processes for the genes with distinct tissue- (left) and stage-specific activity patterns (middle and right)

We then compared the reconstructed EPIs across the 439 cell and tissue types (Fig. 3a and “Methods”). To this end, we hierarchically clustered the reconstructed EPIs based on the overlap of their linkages (i.e., edges in the networks) between each sample. As expected, functionally related and/or biologically similar lineages were consistently grouped together at both the bulk tissue and the cellular level (Additional file 1: Fig. S8 and S9). For instance, the neural cell and tissue types, particularly those from the developmentally similar stages (i.e., fetal, newborn and adult stages), could be generally grouped together (Fig. 3b). By contrast, the clustering of the cell and tissue types based on the promoter activity revealed a higher degree of noise, suggesting that the reconstructed EPIs can better represent the lineage specificity than the promoter activity (Additional file 1: Fig. S10). Furthermore, we identified enhancers and their target promoters that are specifically active in a single group of related samples. We found that the reconstructed interactions between enhancers and promoters exhibited a high degree of specificity within the groups consisting of related samples (Fig. 3c). For instance, the EPIs in the brain were distinct from those in immune cells. Taken together, these results suggested that the reconstructed EPIs are biologically meaningful and could represent their intrinsic regulatory components in specific samples.

### CRE activity captures cell/tissue-type-specific enhancer function in human brain

Given that the FANTOM5 samples cover a large number of cell and tissue types from human brain as well as a wide range of whole-body lineages, the data analyzed here have made it possible to systematically compare the brain enrichment of CREs and their interactions with the whole-body tissue specificity (Methods). In total, 54 different neural cell and tissue types (6 neural cell types and 48 brain tissue types) were included, most of which are from specific brain regions. Notably, we have samples from diverse brain regions in different developmental stages, i.e., fetal (*n* = 4), newborn (*n* = 14), and adult (*n* = 30) brains. We partitioned the enhancers and promoters into different categories based on their relative activity between brain and non-brain cell/tissue types, as well as different developmental stages (i.e., comparison across fetal, newborn and adult from the brain) (Additional file 2: Table S5 and Table S6).

We found that there are more promoters than enhancers with elevated activity in the brain samples, and brain-specific enhancers exhibit higher activity compared to other tissues (Additional file 1: Fig. S11 and S12). While for the stage specificity, there are much more enhancers and promoters with elevated activity in the fetal and newborn brain than in the adult brain, highlighting actively gene expression regulation in the early stage of brain development, which is consistent with the regulatory peak in perinatal stage that involved in morphine addiction, cell proliferation, growth, and migration pathways [79].

We then investigated whether the enhancers with distinct tissue and stage specificity were likely to be of functional significance using measure of conservation (Fig. 3d). We observed that both the sets of enhancers with elevated activity in the brain samples (*P* < 2.22e − 16, Wilcoxon test) and broad activity in the samples (*P* < 2.22e − 16, Wilcoxon test) are more conserved than those with elevated activity in non-brain samples, suggesting that these enhancers might play key roles in the functionality across the whole body, particularly in human brain. In addition, the enhancers with elevated activity in the fetal brain show higher degree of conservation than those with elevated activity in the adult (*P* = 0.04, Wilcoxon test) and newborn brain (*P* = 4.40e − 7, Wilcoxon test), which is in line with our previous finding showing that active enhancer regulation is particularly important in the early developmental stage of human brain.

To identify the transcription factors (TFs) that potentially mediate tissue- and stage-specific gene regulation, we calculated the overlap of footprinted transcription factor binding sites with the enhancers of distinct tissue- and stage-specific activity patterns. It should be noted that there is extensive sharing of binding preference between the TFs within TF families [80], so it is often not possible to determine which TF(s) from a TF family binds to a given region. Still, we found that many TFs exhibited distinct enrichment patterns for different sets of enhancers. The enhancers with elevated activity in newborn brains contain the most bound TFs (213 TFs) compared to those in fetal (128 TFs) and adult (46 TFs) brains (Additional file 1: Fig. S13). Here, we highlighted some TF genes with top binding potential on the enhancers with different tissue and stage specificity (Fig. 3e). There is previous evidence to support some of the predicted TF associations to brain development. In particular, we noted that TFs RFX1, RFX2, RFX5, and CTCF that function in brain development [81, 82] show significant enrichment in fetal and newborn specific enhancers; TF EGR1 is a major mediator and regulator of synaptic plasticity and neuronal activity [83]; SOX gene family (e.g., SOX2, SOX3, SOX4, SOX6, SOX15, and SOX17) is involved in the regulation of embryonic development, especially in the developing nerve cells of CNS and the transition of cerebellar epithelial granule cells to a migratory state [84, 85]; TFs FRA1, FOSL2, and ATF3 which are key regulators for inflammatory and immune pathways [81], exhibiting a significant enrichment in enhancers of whole stages.

Finally, we implemented the gene function enrichment analysis to examine the overlap of their promoters in different tissue- and stage-specific activity patterns with previously annotated genes involved in biological processes (Fig. 3f). We found that the genes with specific brain-associated activity or regulatory patterns are significantly enriched in relevant biological functions. For example, the genes that are specifically elevated and regulated in brain instead of in non-brain are significantly enriched in axon development, axongenesis, and neurotransmitter secretion; the target genes of enhancers elevated in fetal brain are enriched in mitochondrial translation and expression, while the elevated genes in fetal brain are enriched in cellular response to chemokine; the genes elevated in newborn brain are enriched in protein localization to endoplasmic reticulum; the genes elevated in adult brain are enriched in ensheathment of axon and neurons. *Associating cell types and developmental stages to brain disorders using heritability enrichment of CREs.*

Given that most disorder- and behavior/cognition-associated common genetic variants affect the gene regulation of gene expression rather than protein–protein interaction and/or protein structure [1, 37, 86], we then used an LD score partitioned heritability approach [35] to explore the overlap of active CREs from the diverse brain cell types and regions with genetic variants associated with traits, while correcting for the genomic background (Methods). In total, we included 17 brain disorders and behavioral-cognitive phenotypes, consisting of 5 neurological disorders (including Alzheimer’s disease (AD), amyotrophic lateral sclerosis (ALS), epilepsy, multiple sclerosis (MS), and Parkinson’s disease (PD)), 8 psychiatric disorders (including attention deficit hyperactivity disorder (ADHD), anxiety, autism spectrum disorder (ASD), alcohol use disorder (AUD), bipolar disorder (BIP), obsessive–compulsive disorder (OCD), schizophrenia (SCZ), and Tourette’s syndrome (TS)), and 4 behavioral-cognitive phenotypes (including insomnia, intelligence, neuroticism, and risk behavior) (Additional file 2: Table S7).

**Fig. 4:**
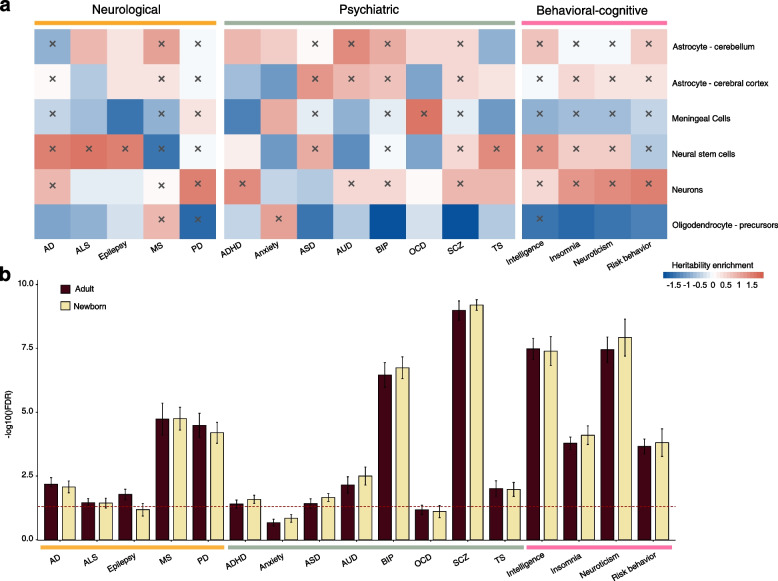
Partitioned heritability enrichment of CREs from different brain cell and tissue types across diverse brain disorders and behavioral-cognitive phenotypes. **a** Heatmap showing the partitioned heritability enrichment of genetic variants overlapping CREs from brain cell types across different brain disorders and behavioral-cognitive phenotypes. The normalized partitioned heritability enrichment scores under the significance level of 5% were labeled with “ × ”. **b** Histogram showing the partitioned heritability enrichment of genetic variants overlapping CREs from adult and newborn brain tissues across different brain disorders and behavioral-cognitive phenotypes. The red dashes line indicates the FDR at the significance level of 5%. AD, Alzheimer’s disease; ADHD, attention deficit hyperactivity disorder; ALS, amyotrophic lateral sclerosis; ASD, autism spectrum disorder; AUD, alcohol use disorder; BIP, bipolar disorder; MS, multiple sclerosis; OCD, obsessive–compulsive disorder; PD, Parkinson’s disease; SCZ, Schizophrenia; TS, Tourette’s syndrome

First, we analyzed the partitioned heritability enrichment of CREs in six different neural cell types (Fig. 4a and Additional file 2: Table S8). Notably, FANTOM5 dataset had limited representation of important neural cell types such as microglia, oligodendrocytes, and radial glia, which may hinder the interpretation of the results. Despite the well-established roles of glia in AD pathogenesis, we were unable to show these enrichment patterns in our results due to the absence of glia in the dataset [87–89]. Interestingly, our findings suggested that AD exhibits strong enrichment in neural stem cells, which give rise to various neuronal and glial cell types in the brain. This enrichment could be attributed to the lineage and functional connections between neural stem cells and glia [90]. In addition to AD, we found that ALS and epilepsy exhibited enrichment in the neural stem cells and astrocytes [91, 92], indicating that, likewise, glia cells such as astrocytes and their progenitor lineage, neural stem cells, might be critical for the pathogenesis of these two neurological disorders. We also found that PD exhibited strongest enrichment in the neurons, consistent with the pathogenesis of dopamine-producing neurons in PD [93, 94].

For most psychiatric disorders and behavioral-cognitive phenotypes, as expected, we observed significant enrichment in neurons, while we also found pervasive enrichment in astrocytes, highlighting the potential functionality of astrocytes in the regulation of behavior and cognitive ability [95–97]. For example, insomnia could be influenced by the dysregulation of Ca^2+^ level in astrocytes, and insomnia-related genetic variants are significantly enriched in the claustrophobic neurons [55, 98]. Interestingly, our results also suggested that meningeal cells may have crucial roles in the pathogenesis of anxiety and OCD. However, we have very limited knowledge on the functions of meningeal cells in human brain, thus the underlying mechanism of meningeal cells in these specific types of anxiety disorders is still unclear.

We then investigated the heritability enrichment of CREs from diverse newborn and adult brain regions across the brain disorders and behavioral-cognitive phenotypes (Fig. 4b, Additional file 1: Fig. S14 and Additional file 2: Table S8). Psychiatric disorders (e.g., ADHD, anxiety, ASD, AUD, BIP, and SCZ) showed higher heritability enrichment in the newborn brain compared to the adult brain; while for most neurological disorders (e.g., AD, epilepsy and PD), we did not observe these patterns. This is consistent with previous studies showing that psychiatric disorders exhibit epigenomic and transcriptomic dysregulations in the early developmental stages, regions, and cell types and thus have been considered as a group of neurodevelopmental disorders [99, 100]. Interestingly, as what we observed for psychiatric disorders, the behavioral-cognitive phenotypes (e.g., insomnia, neuroticism and risk behavior) also exhibit higher heritability enrichment in the newborn brain, suggesting putative significant genetic risk correlation between psychiatric disorders and behavioral-cognitive phenotypes [101].

Jointly, these results highlight the importance of studying the cell-type- and stage-specific gene regulation to properly dissect the genetic architecture of brain disorders and behavioral-cognitive phenotypes. More importantly, our results reveal novel heritability enrichment patterns for some cell types (e.g., meningeal cells) and developmental stages (e.g., newborn) that have not been well characterized before.

### Prioritizing genes associated with brain disorders and behavioral-cognitive phenotypes in diverse cell types and regions based on EPIs

**Fig. 5:**
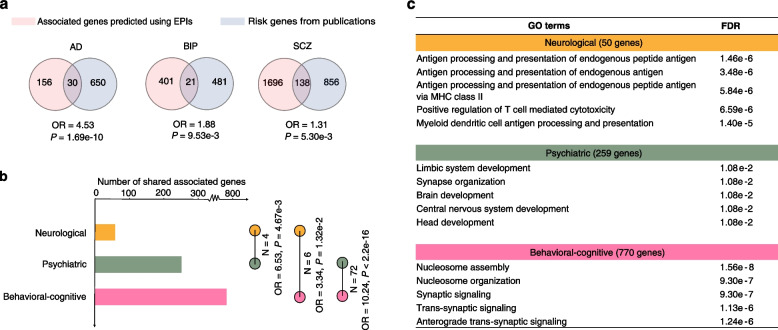
Prioritized genes associated with brain disorders and behavioral-cognitive phenotypes. **a** Overlap between the prioritized disorder-associated genes based on EPIs and known risk genes for three brain disorders. The *P*-values were estimated using Fisher’s exact test. AD, Alzheimer’s disease; BIP, bipolar disorder; SCZ, schizophrenia. **b** Bar plot showing the number of associated genes that are shared within each category of brain disorders and behavioral-cognitive phenotypes (left), as well as the number of shared associated genes between different categories (right). The *P*-values were estimated using Fisher’s exact test. **c** The most enriched biological processes (top five) of the shared associated genes for each category of brain disorders and behavioral-cognitive phenotypes

Next, we aim to prioritize the genes associated with brain disorders and behavioral-cognitive phenotypes by using the reconstructed EPIs in the diverse brain cell types and regions. To this end, we applied our nMAGMA algorithm [62] that can better infer risk genes by assigning the distal noncoding SNPs [6] to their target genes based on the predicted EPIs (Methods); thus, we prioritized the genes associated with the brain disorders and behavioral-cognitive phenotypes for each cell and tissue type from human brain (provided on our supplementary website; URL: https://soulnature.github.io/brainepl). Here, we highlighted that the associated genes predicted by our approach exhibit significant overlap with the previously known risk genes for SCZ (*P* = 5.30e − 3, Fisher’s exact test), BIP (*P* = 9.53e − 3, Fisher’s exact test), and AD (*P* = 1.69e − 10, Fisher’s exact test) (Fig. 5a and Additional file 2: Table S9), confirming the utility of our reconstructed EPIs. Also, a large proportion of previously known risk genes cannot be rediscovered by our analysis, partially due to that here we only considered the *cis*-regulatory mechanism in prioritizing the risk genes; however, the pathogenesis of these complex brain disorders are involved in multiple layers of gene regulation such as post-transcriptional regulation and post-translational regulation [102–105].

We then systematically predicted the associated genes for all the 17 brain disorders and behavioral-cognitive phenotypes, among which intelligence (2466 genes) and SCZ (1908 genes) show the most associated genes, which is in line with their high heritability [106]. Some previously known risk genes of brain disorders could be successfully prioritized by nMAGMA [62], such as NKAPL and PBX2 for SCZ [107, 108], and MAPT for PD [109]. Notably, among the 17 different brain disorders and behavioral-cognitive phenotypes assessed here, we did not find any significant associated genes for three disorders including anxiety, OCD, and TS, which might result from a lack of power in the current GWAS data, or the limited number of EPIs in the brain cell and tissue types for disorders.

### Widespread pleiotropy of the associated genes between psychiatric disorders and behavioral-cognitive phenotypes

Given shared heritability across different brain disorders and behavioral-cognitive phenotypes [101], we examined shared associated genes of different brain disorders and/or behavioral-cognitive phenotypes to identify their common molecular mechanisms. In total, we found 50, 259, and 770 associated genes that are shared by at least two traits within each category of neurological disorders, psychiatric disorders, and behavioral-cognitive phenotypes, respectively (Fig. 5b and Additional file 2: Table S10). Furthermore, we found that these shared associated genes exhibit significant overlap between different categories of brain disorders and behavioral-cognitive phenotypes, indicating strong pleiotropy of these associated genes and their putative common mechanisms (Fig. 5b). Interestingly, *FOXP2*, a crucial gene for speech evolution and language disorders [110, 111], was identified as a pleiotropic gene of psychiatric disorders and behavioral-cognitive phenotypes including ADHD, insomnia, intelligence, neuroticism, and risk behavior.

The shared associated genes of different categories of brain disorders and behavioral-cognitive phenotypes exhibit distinct enriched biological functions (Additional file 2: Table S11). The shared associated genes of neurological disorders are involved in immune-associated functions (Fig. 5c), such as antigen processing and presentation of endogenous antigen, supporting the hypothesis that neuroinflammation could be crucial for the pathophysiology of multiple neurological disorders [112, 113]. While those shared associated genes of psychiatric disorders and behavioral-cognitive phenotypes show enriched biological functions of gene regulation, synaptic function, neuronal development (Fig. 5c), which is in line with the biological functions of synapses in the regulation of memory, perception, attention, and learning [114–117] and their established associations with complex psychiatric disorders [118, 119]. Furthermore, we confirmed that the psychiatric disorders and behavioral-cognitive phenotypes showed more enrichment in synapse-associated processes than neurological disorders by using the SynGO annotations [120] (Additional file 1: Fig. S15).

### Expression signatures of the genes associated with brain disorders and behavioral-cognitive phenotypes

**Fig. 6:**
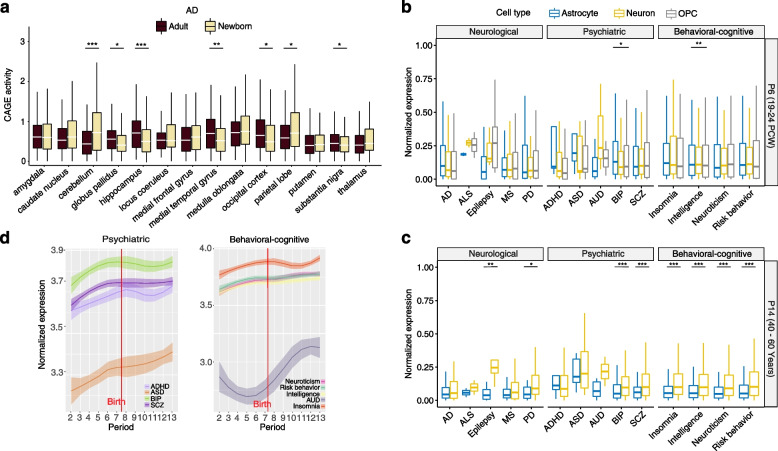
Expression dynamics of the associated genes of brain disorders and behavioral-cognitive phenotypes. **a** The CAGE activity profiles of the AD-associated genes in diverse brain regions. For each brain region, we showed the expression levels of the associated genes at the newborn and adult tissues, respectively. *** *P* < 0.001; ** *P* < 0.01; * *P* < 0.05; Wilcoxon test. AD, Alzheimer’s disease. **b,c** Expression profiles of the genes associated with diverse disorders and behavioral-cognitive phenotypes from different brain single-cell types at the fetal (**b**) and adult (**c**) stage. The gene expression data of brain single cells were collected from STAB database [70]. We used single-cell datasets from 19–26 PCW and 40–60 years in the analysis at the fetal and adult stage, respectively. *** *P* < 0.001; ** *P* < 0.01; * *P* < 0.05; ANOVA test. PCW, postconceptional weeks. OPC, oligodendrocyte progenitor cell. **d** Developmental expression trajectories of the genes associated with disorders and behavioral-cognitive phenotypes. The gene expression data were collected from Zhu et. al [8]. The LOESS plots show smooth curves with 95% confidence bands. P2, 8 ≤ Age < 10 PCW; P3, 10 PCW ≤ Age < 13 PCW; P4, 13 PCW ≤ Age < 16 PCW; P5, 16 PCW ≤ Age < 19 PCW; P6, 19 PCW ≤ Age < 24 PCW; P7, 24 PCW ≤ Age < Birth; P8, Birth ≤ Age < 6 months; P9, 6 months ≤ Age < 1 year; P10, 1 ≤ Age < 6 years; P11, 6 ≤ Age < 12 years; P12, 12 ≤ Age < 20 years; P13, 20 ≤ Age < 60 years. PCW, postconceptional weeks

To systematically evaluate the spatiotemporal expression patterns of the genes that associate with brain disorders and behavioral-cognitive phenotypes, we leveraged large brain transcriptomic datasets to assess in which brain regions or cell types and developmental stages, these associated genes show higher expression level. First, we examined the CAGE activity level of the associated genes at different developmental stages. Consistent with our previous results from the heritability enrichment, we found that the genes associated with neurological disorders generally exhibited higher activity in adult brain than in newborn brain, while the associated genes of psychiatric disorders and behavioral-cognitive phenotypes exhibited higher activity in newborn brain. For instance, the genes associated with AD exhibited significantly higher activity in the adult brain regions such as hippocampus (*P* = 4.30e − 5, Wilcoxon test) and medial temporal gyrus (*P* = 1.70e − 3, Wilcoxon test), which have been well characterized in the pathogenesis of AD [121–123] (Fig. 6a). Similarly, for PD, the associated genes exhibited significantly higher activity in the adult brain regions such as globus pallidus (*P* = 3.23e − 3, Wilcoxon test) and medial temporal gyrus (*P* = 0.02, Wilcoxon test) (Additional file 1: Fig. S16). In the case of ADHD, the associated genes showed higher activity in the fetal locus coeruleus (Additional file 1: Fig. S17). Although it has been clear that locus coeruleus is the primary source of norepinephrine, for which the dysfunction has been implicated in the development of ADHD [124, 125], our results suggest that the fetal stage of locus coeruleus may be more critical than the adult stage in the pathogenesis of ADHD. Additionally, we noticed that for SCZ and BIP, their associated genes showed higher activity in the adult stage of occipital cortex compared to the fetal stage. Notably, we did not observe higher activity of the associated genes in substantia nigra in adult brain, possibly due to the limited sample size of substantia nigra from the FANTOM5 dataset. Taken together, our results not only confirmed the well-established developmental stages and brain regions most specifically affected by the brain disorders, but also highlight the putative associations between these brain disorders and some brain regions that have not been well characterized (e.g., globus pallidus and medial temporal gyrus).

In addition to the disorder-centric analysis, we also performed analysis in region-centric manner to determine their most relevant brain disorders and/or behavioral-cognitive phenotypes, as well as developmental stages, in which the associated genes have higher expression levels. We showed the results from four different brain regions including cerebral cortex, hippocampus, occipital lobe, and temporal lobe (Additional file 1: Fig. S18). As expected, for psychiatric disorders and behavioral-cognitive phenotypes, their associated genes generally exhibit higher expression levels in the fetal brain than in the newborn and adult brain, which is consistent with previous studies showing that altered gene regulation in the early prenatal brain could be more susceptible to various psychiatric disorders [65, 126, 127].

Furthermore, we explored the expression of the associated genes in astrocytes, neurons, and oligodendrocyte progenitor cells (OPCs) from prenatal (period 6, i.e., 19 PCW-22 PCW) and adult (period 14, i.e., 40–60 years) stage using single-cell transcriptomic data from our STAB database [70]. The associated genes show distinct expression patterns in different cell types and developmental stages (Fig. 6b, c). Notably, the associated genes generally show higher expression in neurons than in astrocytes in adult stage instead of in fetal stage. We also investigated the expression of the associated gene in three cell types during lifespan, and found that most brain disorders and behavioral-cognitive phenotypes have similar patterns of developmental trajectories in OPCs and astrocytes, with an activity peak in the mid-infancy (Additional file 1: Fig. S19).

Finally, we analyzed the expression trajectories of the associated genes of psychiatric disorders and behavioral-cognitive phenotypes across the whole lifespan (Fig. 6d and Additional file 2: Table S12). We found that genes associated with SCZ and BIP show similar expression dynamic pattern, with a gradual increase during the prenatal period and a relative stable trend after birth, consistent with previous studies showing the shared genetic basis of these two complex brain disorders [101, 128, 129]. While the genes associated with neuroticism, intelligence, and risk behavior share the same pattern, which exhibits two peaks at period 6 (i.e., 19 PCW-22 PCW) and period 11 (i.e., 8–11 years). Previous studies suggest that both periods are important for brain reconstitution and fast development: period 6 (in mid-gestation stage) is important for axonogenesis and dendritic arborization [71], and period 11 (in adolescence stage) represents a key stage for the rapid development of brain cortical regions that are responsible for higher cognitive and emotional functions [130]. Overall, we systematically characterized the spatiotemporal expression dynamics of the associated genes, and highlighted the putative crucial brain regions and developmental stages for the diverse brain disorders and behavioral-cognitive phenotypes.

## Discussion

In this study, we used large-scale enhancer and promoter activity (CAGE) data to reconstruct EPIs in a wide range of cell and tissue types, and systematically examined the activity and architecture in diverse neural cell and tissue types. By using the active CREs and their EPIs in each neural cell and tissue type, we explored the heritability enrichment of different brain disorders and behavioral-cognitive phenotypes, expanding the neural cell types, brain regions, and developmental stages assessed. We found that psychiatric disorders and neurological disorders generally show enriched heritability in fetal and adult brains, respectively. For each brain disorder and behavioral-cognitive phenotype, a set of associated genes were prioritized based on the EPIs from neural cell and tissue types. We show that the pleiotropic associated genes of psychiatric disorders and behavioral-cognitive phenotypes are enriched in synapse-associated functions, while the pleiotropic associated genes of neurological disorders are enriched in immune-related functions. A key benefit of our analysis is its ability to utilize the power from the distal SNPs with weak GWAS significance. This allows researchers to predict the associated genes in a cell and tissue-specific manner and gain novel insights into the genetic risk factors of these complex disorders.

In contrast to actively expressed genes, CREs are more numerous and exhibit activity in specific cell and tissue type, and their sequences can provide clues about the regulatory programs that drive cell state specification [131, 132]. By combining the activity information of the enhancers with the activity measurements of the promoters based on CAGE technology, we are able to systematically reconstruct EPIs for a wide range of cell and tissue types, including numerous ones from human brain. We anticipate that future studies could leverage our data to experimentally demonstrate the detailed regulatory mechanisms that govern the development of human brain, the specification of neurons and glial cells, and pathogenesis of brain disorders. Notably, in our analysis, we cannot separate the active enhancers and poised enhancers [133, 134] by using only the CAGE data from FANTOM5 project. Thus, our current results failed to reveal the state transition of enhancers during brain development and its impact on the expression of their target promoters.

Most existing efforts to link brain disorder risk to transcriptomic or epigenomic dysregulation have primarily used dissected brain tissues (e.g., cerebral cortex and hippocampus) or isolated cells (e.g., neurons and astrocytes) restricted in specific developmental stages (e.g., early fetal or adult period), providing insufficient insights into gene regulation at a cell-type-specific and spatiotemporal resolution. Broader insights into *cis*-regulatory mechanisms in the human brain could be gained by studying more brain regions and different developmental stages. By reusing the public FANTOM5 data [27], we have enabled comprehensive identification of the genes associated with brain disorders and behavioral-cognitive phenotypes in diverse brain regions, as well as across the fetal, newborn, and adult stages. Moreover, we show that the associated genes identified using our approach show significant overlap with known risk genes of brain disorders. With single-cell epigenomic and transcriptomic data recently become tractable and scalable, it will be especially suited to expand the cell landscape in our study to achieve higher cellular and regional resolution when dissecting the genetic architecture of brain disorders and behavioral-cognitive phenotypes.

The clinical phenomena, such as behavioral change, alterations of consciousness, or loss of motor function, lead to great complications about the classification of brain disorders. One such example includes ASD, which is a highly heterogeneous disorder; its clinical presentations can vary in multiple axes of severity, creating a wide spectrum of symptoms [135]. Understanding the genetic underpinnings for a particular subset of ASD and related phenotypes may inform the search for their biological mechanisms and clinically relevant genetic therapies. Elucidating the extent of heterogeneity for the brain disorders allows for a better-informed picture of functional consequences associated with genetic influences. In such cases, characterizing the specific brain regions and/or developmental stages that exhibit high risk for a particular subtype of the disorders may help identify targets for diagnostics and therapeutics. Expanding the brain regions, developmental stages, and cell types examined in the analysis is critical for the achievement of this aim.

Although we have taken advantage of the rich FANTOM5 and GWAS data, these datasets have their own limitations, which will impart some restrictions on our predictions. For example, the FANTOM5 data are in some instances limited to a few replicates for cell and tissue types and there is possible heterogeneity in some samples. The enhancers identified by the FANTOM5 data show limited overlap with those identified by using large-scale ChIP-seq and ATAC-seq datasets [24]. For the partitioned heritability approach, the GWAS datasets analyzed must have a very large sample size and large SNP heritability, and the brain disorders and traits analyzed must be polygenic. It should be also noted that we did not consider other players such as noncoding RNAs, alternative splicing, and protein interactions, which have been shown to have a role in the pathogenesis of brain disorders [9, 136–139]. These limitations may result in less faithful predictions on the genes associated with the brain disorders and behavioral-cognitive phenotypes.

## Conclusions

In conclusion, we leverage the reconstructed EPIs in diverse neural cell and tissue types to prioritize the genes associated with different brain disorders and behavioral-cognitive phenotypes. We highlight the cell and tissue specificity of the EPIs in human brain, and their power in furthering our understanding of genetic heritability of the brain disorders in different brain cell types, regions, and developmental stages. We analyze the pleiotropy of the associated genes by comparing the sets of prioritized genes for different categories of brain disorders and behavioral-cognitive phenotypes. We additionally show the spatiotemporal activity of the associated genes by integrating with large-scale bulk and single-cell transcriptomic datasets from human brain.

## Supplementary Information

**Additional file 1: Figure S1. **Clustering of 958 samples based on the activity of the CREs revealed corresponding groups of cell and tissue types. **Figure S2.** Enrichment of CAGE-derived enhancers from FANTOM5 project and active enhancers defined by histone modification ChIP-seq datasets. **Figure S3.** Enrichment of CAGE-derived enhancers from FANTOM5 project and active enhancers defined by ATAC-seq/scATAC-seq datasets. **Figure S4.** Number of promoters that have a range number of linked enhancers during EPI reconstruction. **Figure S5.** Enrichment of total *cis*-eQTLs and fine-mapped *cis*-eQTLs in the CREs from reconstructed EPIs. **Figure S6.** Strength of the active EPIs and the remaining inactive EPIs reconstructed in each sample. **Figure S7.** The accuracy of different EPI prediction approaches. **Figure S8.** Clustering of 142 tissue types based on the similarities of their reconstructed EPIs. **Figure S9.** Clustering of 158 primary cell types based on the similarities of their reconstructed EPIs. **Figure S10.** Clustering of 439 tissue types and brain samples based on the similarities of their promoter activity. **Figure S11.** Proportion of tissue-specific promoters that are linked to tissue-specific enhancers. **Figure S12.** Overlap between groups of enhancers and promoters with distinct tissue and stage specificity in human brain. **Figure S13.** The enrichment of TF binding in the enhancers with distinct tissue- (up) and stage-specific activity patterns (bottom). **Figure S14.** Heatmap showing the partitioned heritability enrichment of genetic variants overlapping CREs from brain regions across different brain disorders and behavioral-cognitive phenotypes. **Figure S15.** The most enriched synapse-associated functions of the shared associated genes for each category of brain disorders and behavioral-cognitive phenotypes. **Figure S16.** CAGE activity profiles of the promoters associated with PD in diverse brain regions. **Figure S17.** CAGE activity profiles of the promoters associated with psychiatric disorders in diverse brain regions. **Figure S18.** CAGE activity profiles of the associated genes of diverse brain disorders and behavioral-cognitive phenotypes in specific brain regions. **Figure S19.** Developmental expression trajectories of the associated genes in neural cell types.

**Additional file 2: Table S1. **Source and information of the CAGE datasets and samples from FANTOM5 project. **Table S2. **Number of enhancers, promoters and reconstructed EPI in each cell and tissue type.** Table S3.** Tissue match between the FANTOM5 samples and the cell or tissue types from pcHi-C datasets and GTEx project. **Table S4. **AUPR scores of the reconstructed EPIs. **Table S5.** Enhancers with tissue- and stage-specific activity patterns. **Table S6. **Promoters with tissue- and stage-specific activity patterns. **Table S7.** Source and information of the GWAS summary statistics used in this study. **Table S8.** Partitioned LDSC in the CREs of neural cell and tissue types for different brain disorders and behavioral-cognitive phenotypes. **Table S9.** Collection of known risk genes of 7 different brain disorders. **Table S10.** Associated genes that were shared between different categories of brain disorders and behavioral-cognitive phenotypes.** Table S11.** Enriched biological functions of shared associated genes between different categories of brain disorders and behavioral-cognitive phenotypes. **Table S12.** Spatiotemporal transcriptomic datasets of the human and macaque brains used in this analysis.

## Data Availability

The codes used in the analyses can be downloaded from GitHub (https://github.com/Soulnature/brainepl/). The original public datasets were downloaded as described in “Methods”. The reconstructed EPIs in 439 cell and tissue types and the prioritized genes associated with diverse brain disorders and behavioral-cognitive phenotypes can be downloaded in our supplementary website (https://soulnature.github.io/brainepl).
