# Supplementary material for: Prioritizing genes associated with brain disorders by leveraging enhancer-promoter interactions in diverse neural cells and tissues
Source: Genome Med. 2023 Jul 24;15:56. doi: 10.1186/s13073-023-01210-6 (PMC10364416; doi:10.1186/s13073-023-01210-6)
Supplement: Supplementary file 1 — Additional file 1: Figure S1. Clustering of 958 samples based on the activity of the CREs revealed corresponding groups of cell and tissue types. Figure S2. Enrichment of CAGE-derived enhancers from FANTOM5 project and active enhancers defined by histone modification ChIP-seq datasets. Figure S3. Enrichment of CAGE-derived enhancers from FANTOM5 project and active enhancers defined by ATAC-seq/scATAC-seq datasets. Figure S4. Number of promoters that have a range number of linked enhancers during EPI reconstruction. Figure S5. Enrichment of total cis-eQTLs and fine-mapped cis-eQTLs in the CREs from reconstructed EPIs. Figure S6. Strength of the active EPIs and the remaining inactive EPIs reconstructed in each sample. Figure S7. The accuracy of different EPI prediction approaches. Figure S8. Clustering of 142 tissue types based on the similarities of their reconstructed EPIs. Figure S9. Clustering of 158 primary cell types based on the similarities of their reconstructed EPIs. Figure S10. Clustering of 439 tissue types and brain samples based on the similarities of their promoter activity. Figure S11. Proportion of tissue-specific promoters that are linked to tissue-specific enhancers. Figure S12. Overlap between groups of enhancers and promoters with distinct tissue and stage specificity in human brain. Figure S13. The enrichment of TF binding in the enhancers with distinct tissue- (up) and stage-specific activity patterns (bottom). Figure S14. Heatmap showing the partitioned heritability enrichment of genetic variants overlapping CREs from brain regions across different brain disorders and behavioral-cognitive phenotypes. Figure S15. The most enriched synapse-associated functions of the shared associated genes for each category of brain disorders and behavioral-cognitive phenotypes. Figure S16. CAGE activity profiles of the promoters associated with PD in diverse brain regions. Figure S17. CAGE activity profiles of the promoters associated with psychiatric disorders [file 13073_2023_1210_MOESM1_ESM.docx]

**Supplemental Materials**

**Prioritizing genes associated with brain disorders by leveraging enhancer-promoter interactions in diverse neural cells and tissues**

Xingzhong Zhao^*^, Liting Song^*^, Anyi Yang, Zi-Chao Zhang, Jinglong Zhang, Yucheng T. Yang^†^, Xing-Ming Zhao^†^

**Supplemental Figure**


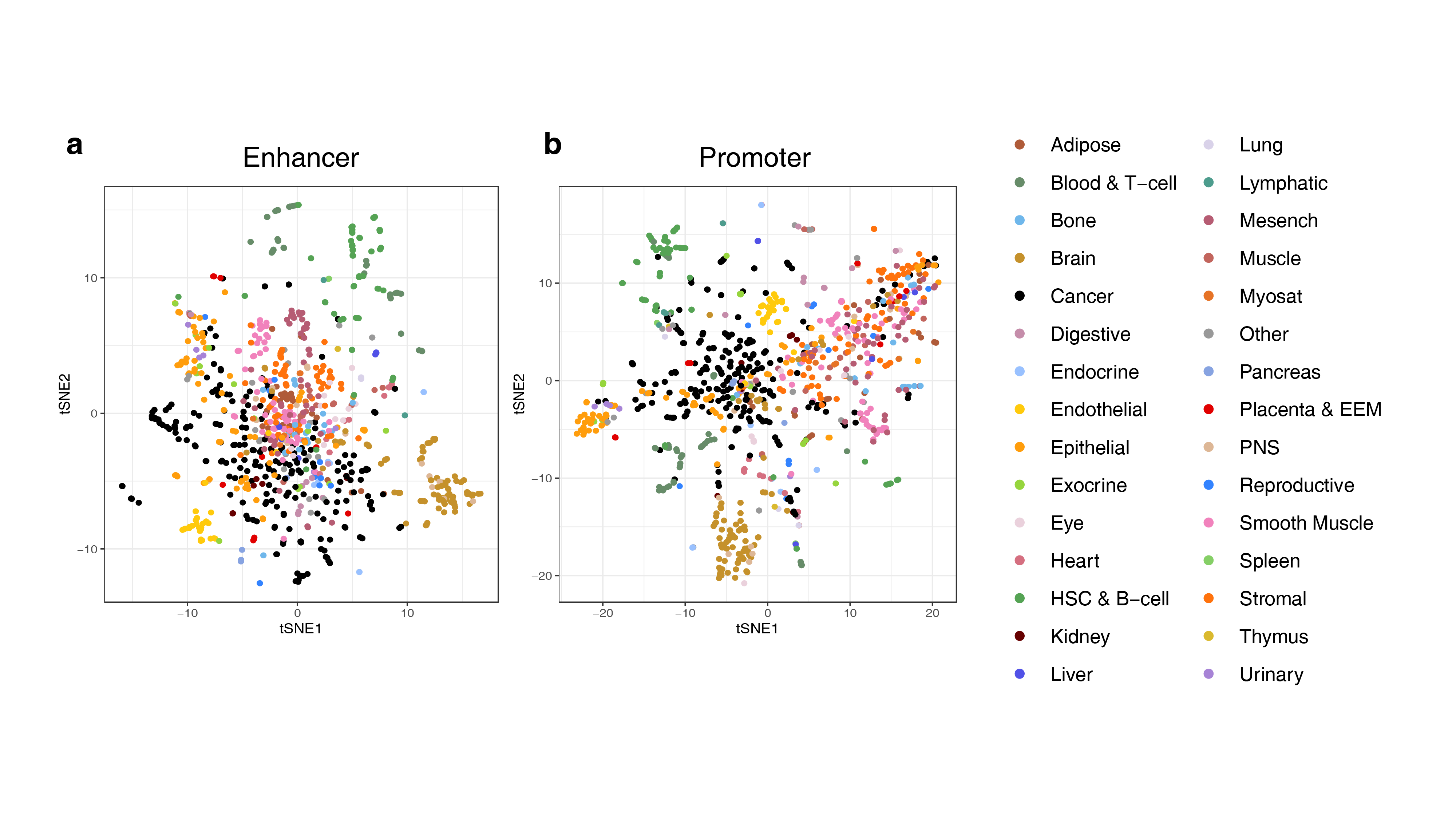


**Figure S1: Clustering of 958 samples based on the activity of the CREs revealed corresponding groups of cell and tissue types.** We performed t-SNE-based clustering using the CAGE activity value of the enhancers (**a**) and promoters (**b**), respectively. Each dot represents a sample colored by its cell and tissue type.

**
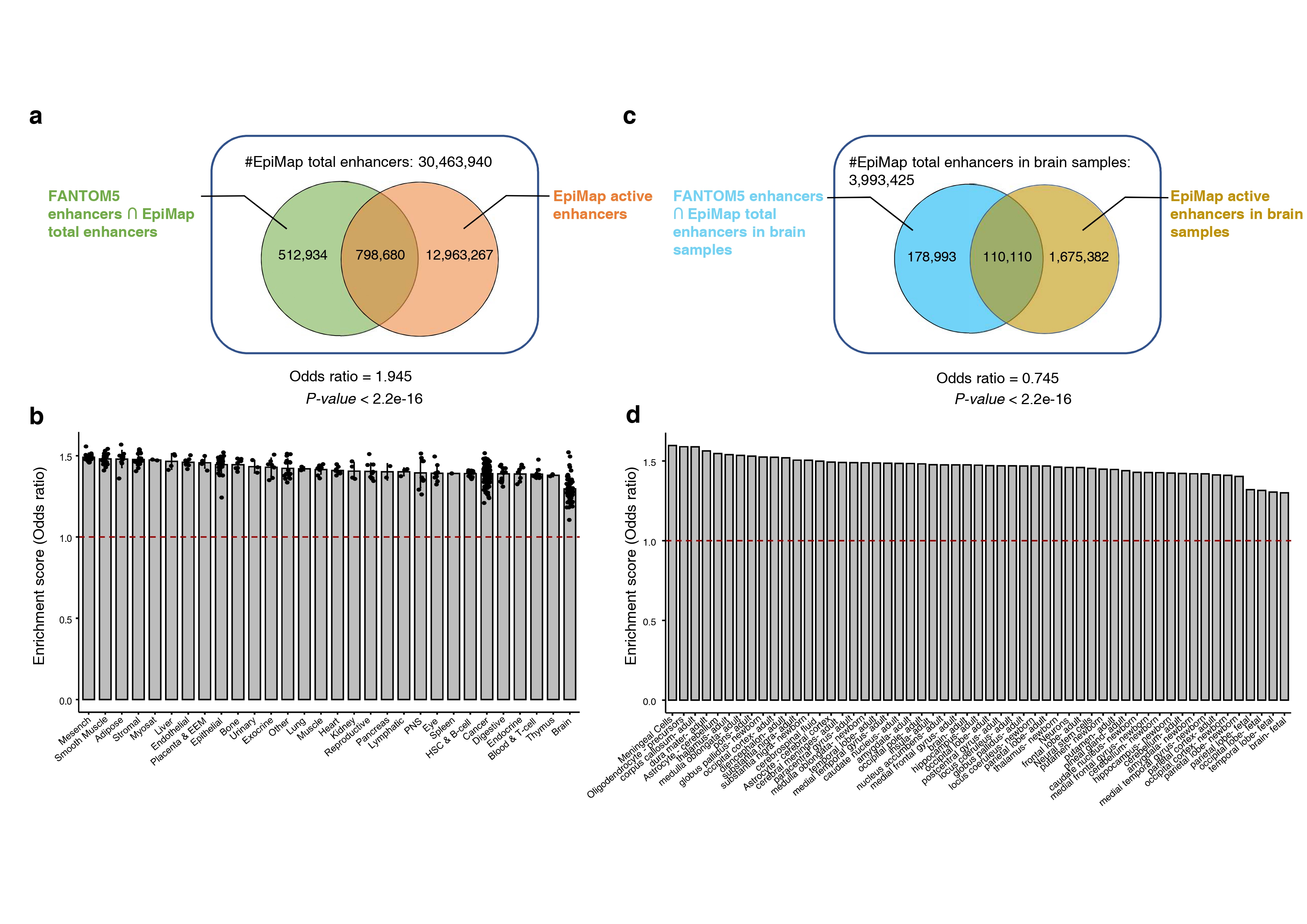
**

**Figure S2: Enrichment of CAGE-derived enhancers from FANTOM5 project and active enhancers defined by histone modification ChIP-seq datasets.** The total enhancers and active enhancers were obtained from EpiMap [1]. (**a**) Overlap between CAGE-derived enhancers from FANTOM5 project and active enhancers in whole-body samples from EpiMap. (**b**) Enrichment scores of CAGE-derived enhancers in a cell/tissue group from FANTOM5 project and the active enhancers in whole-body samples from EpiMap. (**c**) Overlap between CAGE-derived enhancers in brain samples from FANTOM5 project and active enhancers in brain samples from EpiMap. (**d**) Enrichment scores of CAGE-derived enhancers in each brain sample from FANTOM5 project and the active enhancers in brain samples from EpiMap.


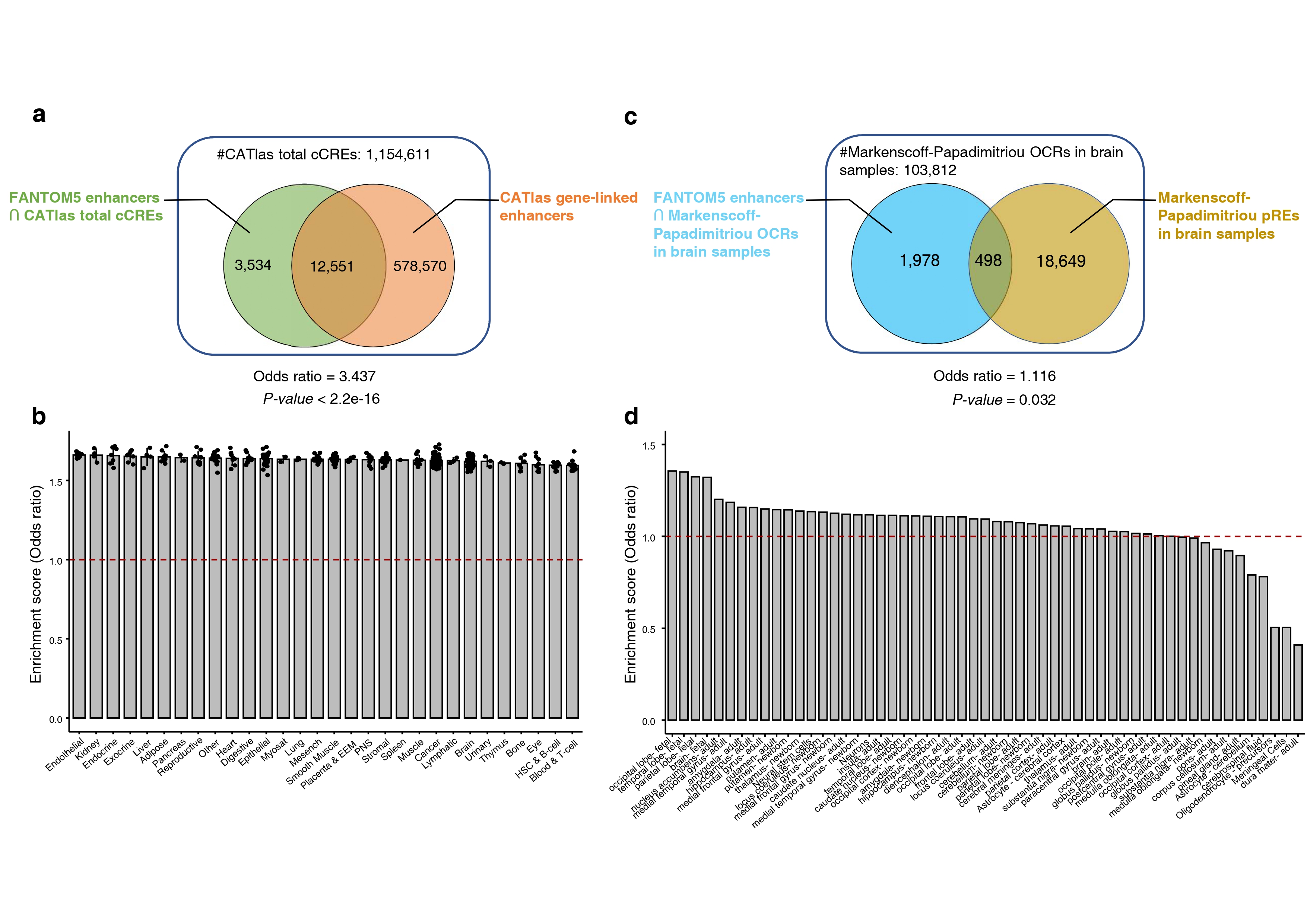


**Figure S3: Enrichment of CAGE-derived enhancers from FANTOM5 project and active enhancers defined by ATAC-seq/scATAC-seq datasets.** The total cCREs and gene-linked cCREs (i.e., active enhancers) were obtained from CATlas [2]. For brain samples, the open chromatin regions (OCRs) and predicted regulatory elements (pRE; i.e., active enhancers) were obtained from Markenscoff-Papadimitriou *et al.* [3]. (**a**) Overlap between CAGE-derived enhancers from FANTOM5 project and active enhancers in whole-body samples from CATlas. (**b**) Enrichment scores of CAGE-derived enhancers in a cell/tissue group from FANTOM5 project and the active enhancers in whole-body samples from CATlas. (**c**) Overlap between CAGE-derived enhancers in brain samples from FANTOM5 project and active enhancers in brain samples from Markenscoff-Papadimitriou *et al.* (**d**) Enrichment scores of CAGE-derived enhancers in each brain sample from FANTOM5 project and the active enhancers in brain samples from Markenscoff-Papadimitriou *et al.*


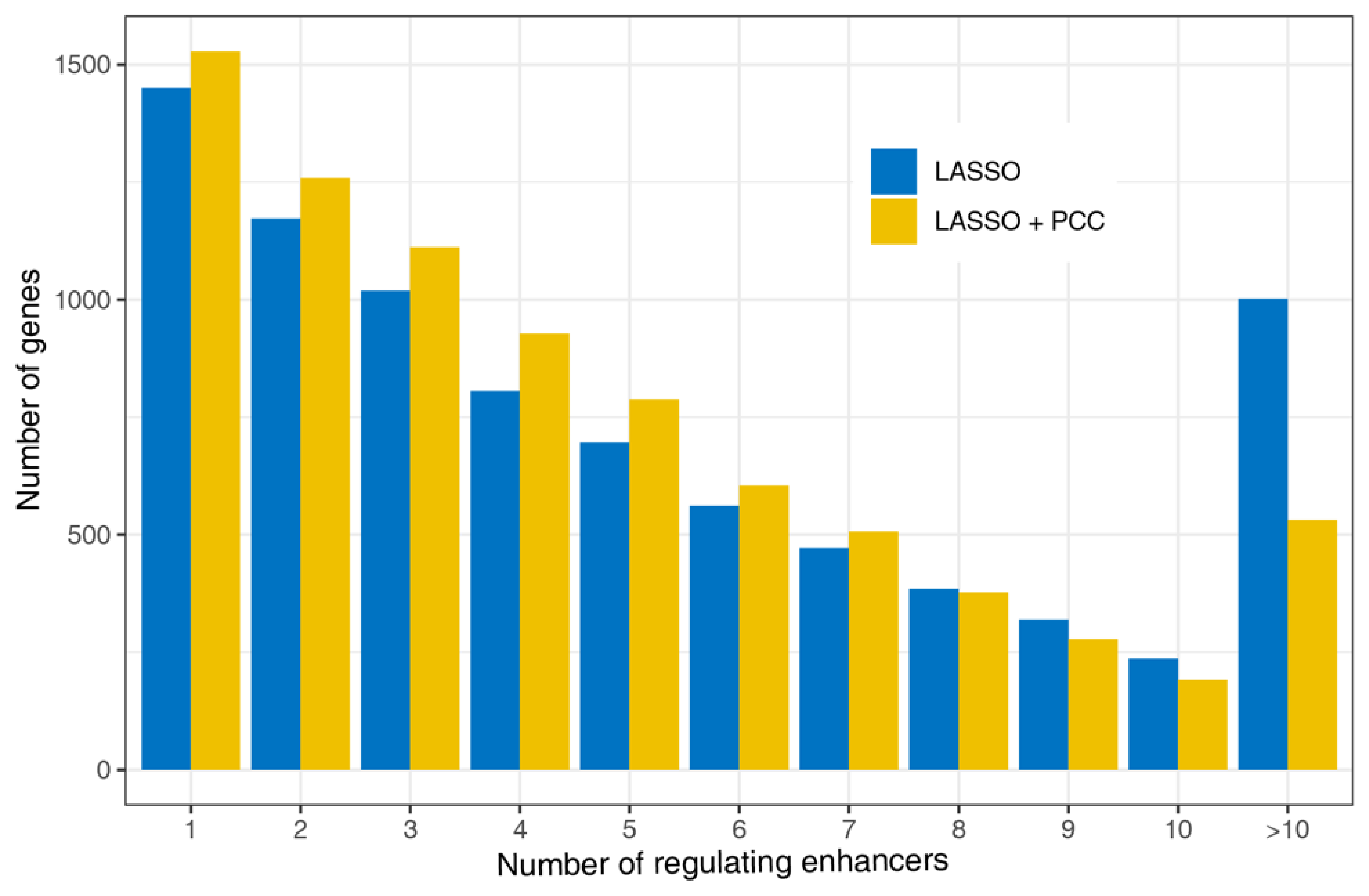


**Figure S4: Number of promoters that have a range number of linked enhancers during EPI reconstruction.** Histogram showing the number of genes that were predicted with a range number of linked enhancers after LASSO regression (blue) and followed by empirical FDR 5% thresholding on Pearson correlation coefficient scores (yellow).


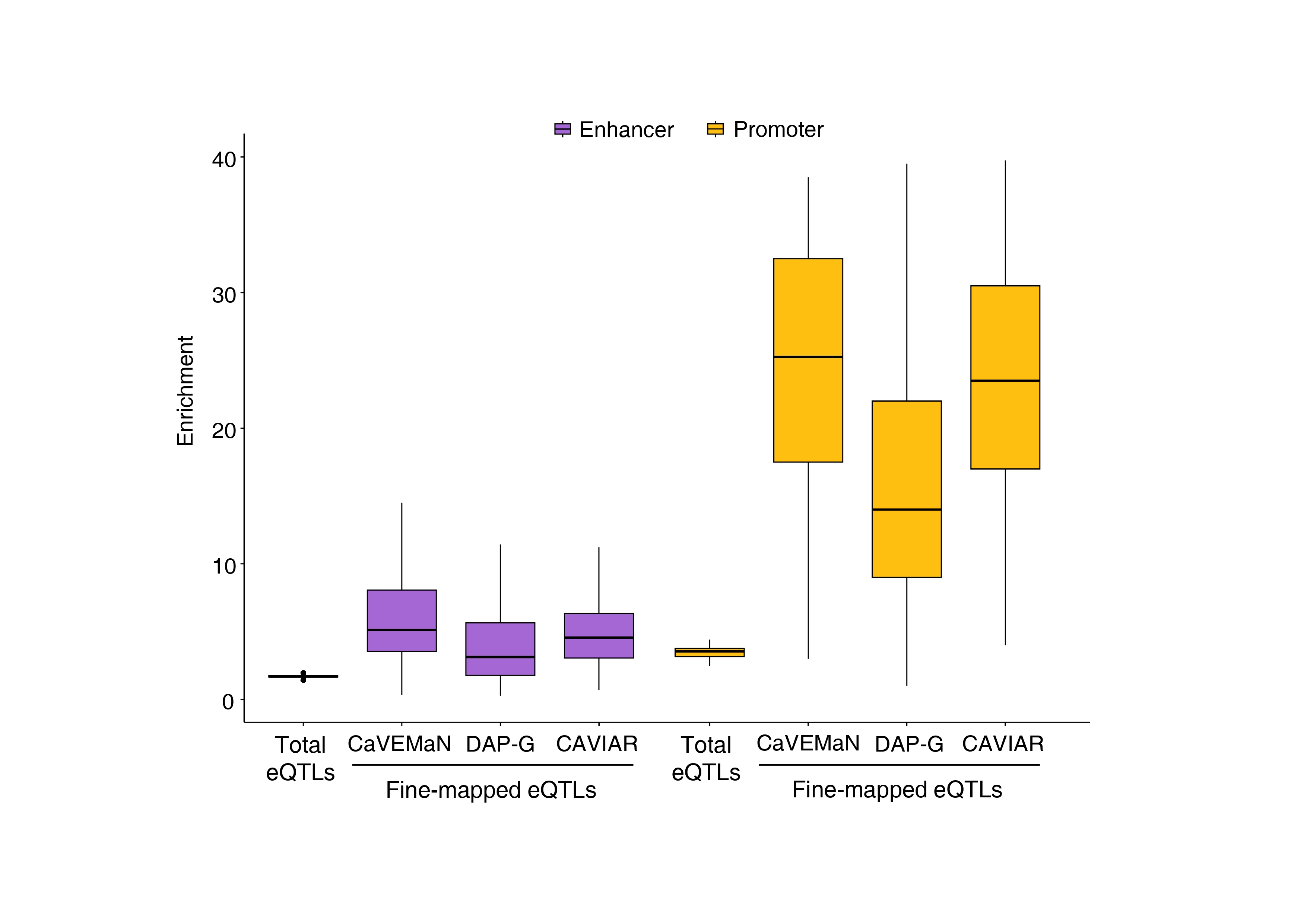


**Figure S5: Enrichment of total *cis*-eQTLs and fine-mapped *cis*-eQTLs in the CREs from reconstructed EPIs.** The fine-mapped *cis*-eQTLs were obtained from the three GTEx fine-mapping *cis*-eQTL catalogs (CAVIAR, CaVEMaN, and DAP-G) and then filtered with a posterior probability >0.8. We calculated odds ratio to estimate the enrichment as we did in the case of total *cis*-QTLs in Figure 2.


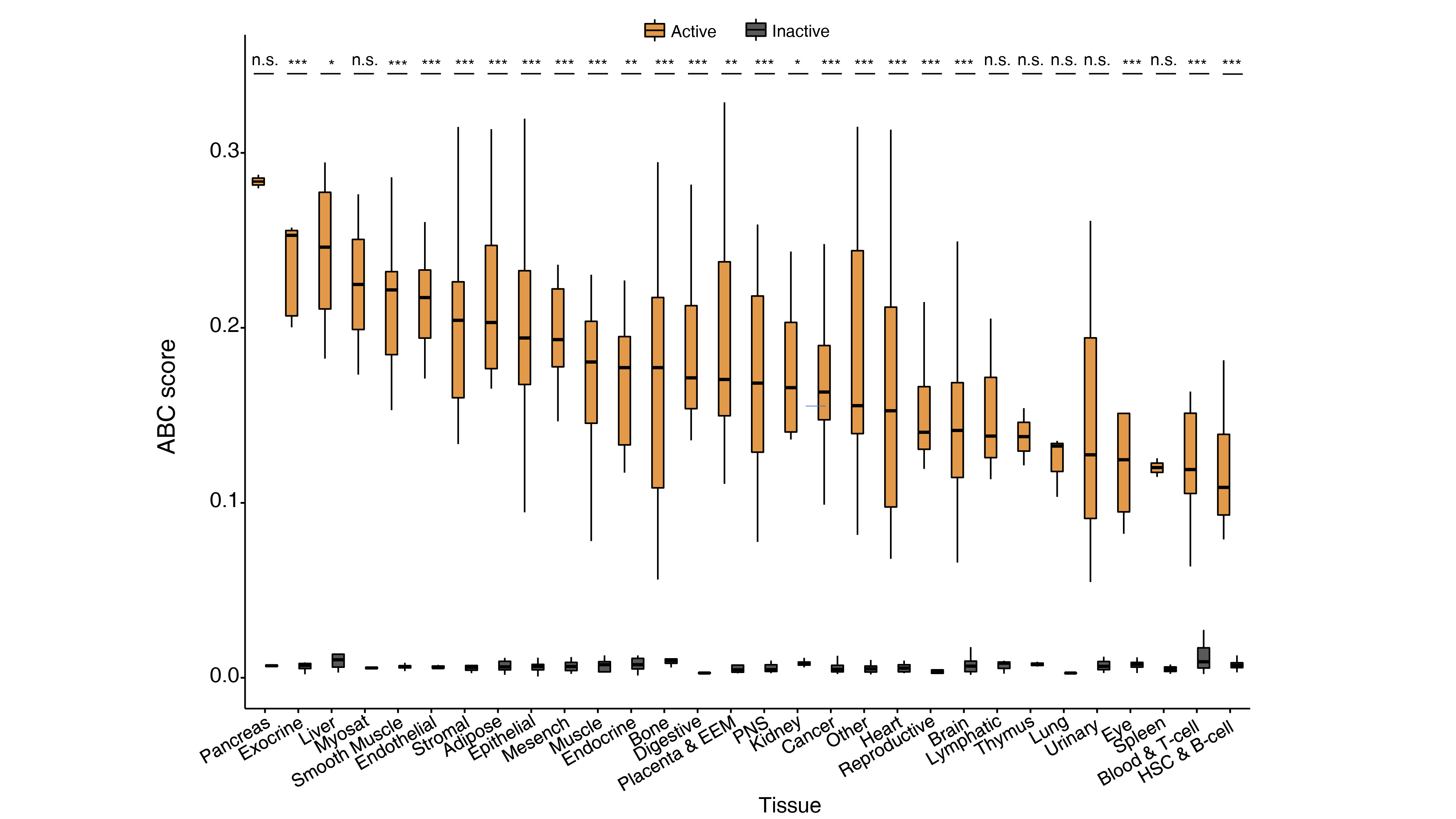


**Figure S6: Strength of the active EPIs and the remaining inactive EPIs reconstructed in each sample.** We quantified the strength of the EPIs in each sample based on the Activity-by-Contact (ABC) model [4]. *** *P* < 0.001; ** *P* < 0.01; * *P* < 0.05; Wilcoxon test.


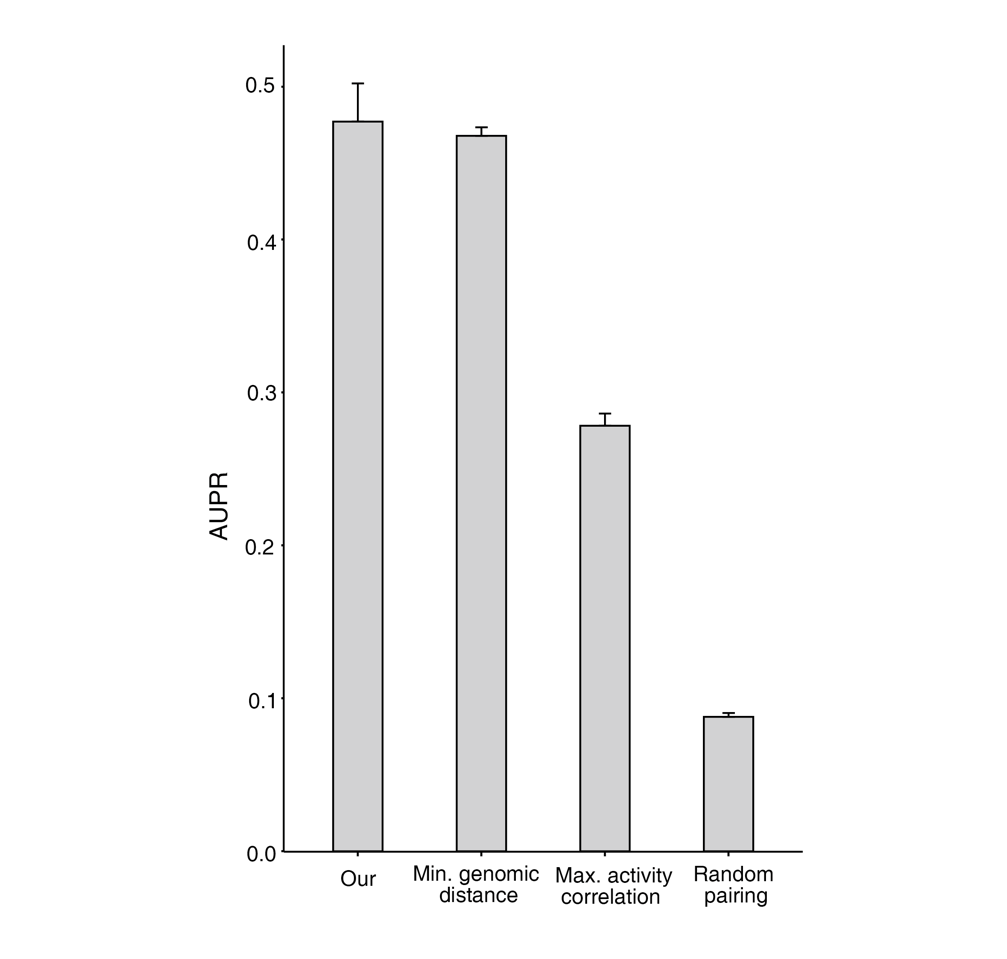


**Figure S7: The accuracy of different EPI prediction approaches.** We estimated the accuracy of the reconstructed EPIs using Area Under the Precision-Recall (AUPR) based on the *cis*-eQTL data from 37 matching and related cell and tissues from the GTEx project [5].

**
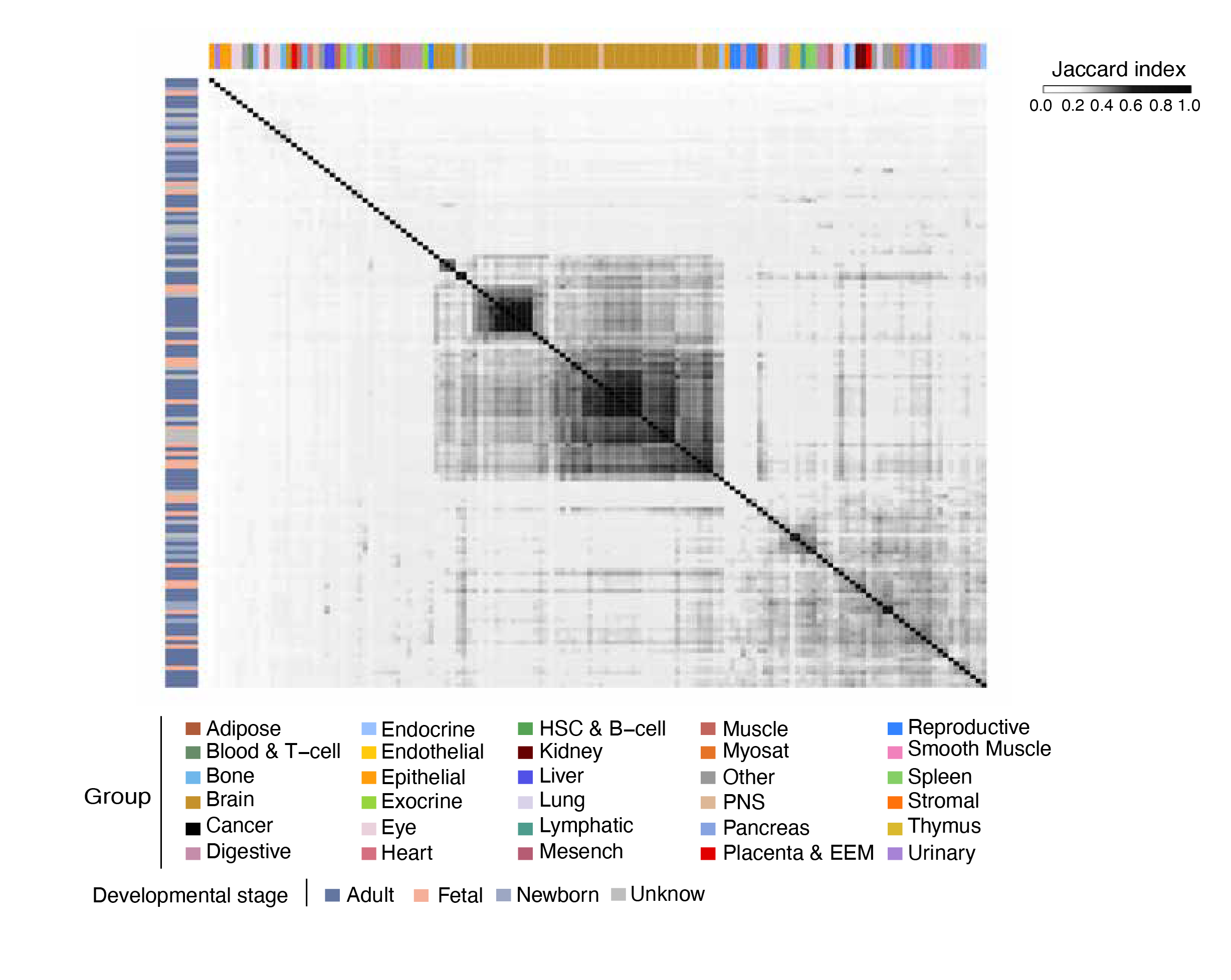
**

**Figure S8: Clustering of 142 tissue types based on the similarities of their reconstructed EPIs.** We used Jaccard index to quantify the overlap of the reconstructed EPIs between each pair of cell and tissue types.


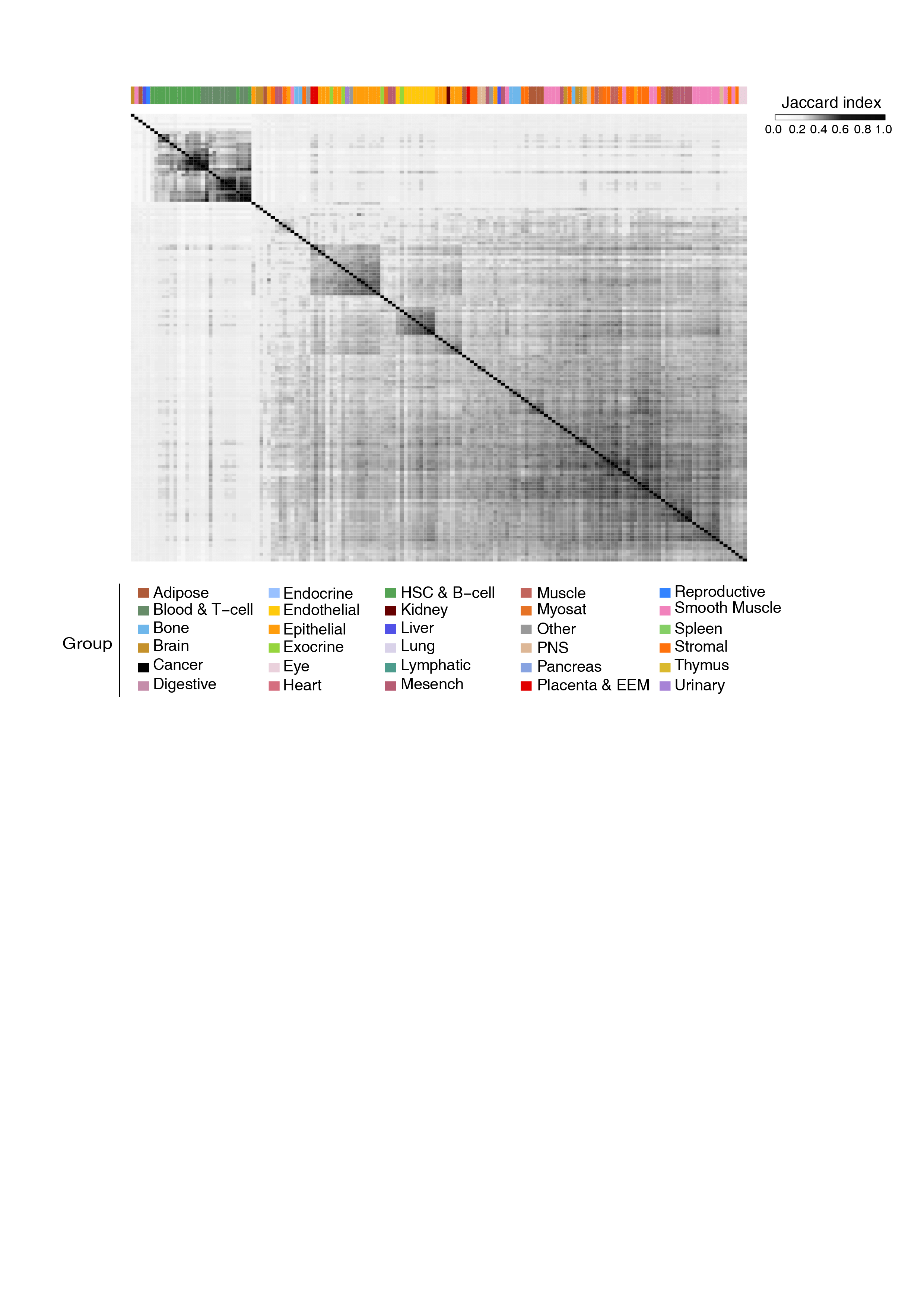


**Figure S9: Clustering of 158 primary cell types based on the similarities of their reconstructed EPIs.** We used Jaccard index to quantify the overlap of the reconstructed EPIs between each pair of cell and tissue types.


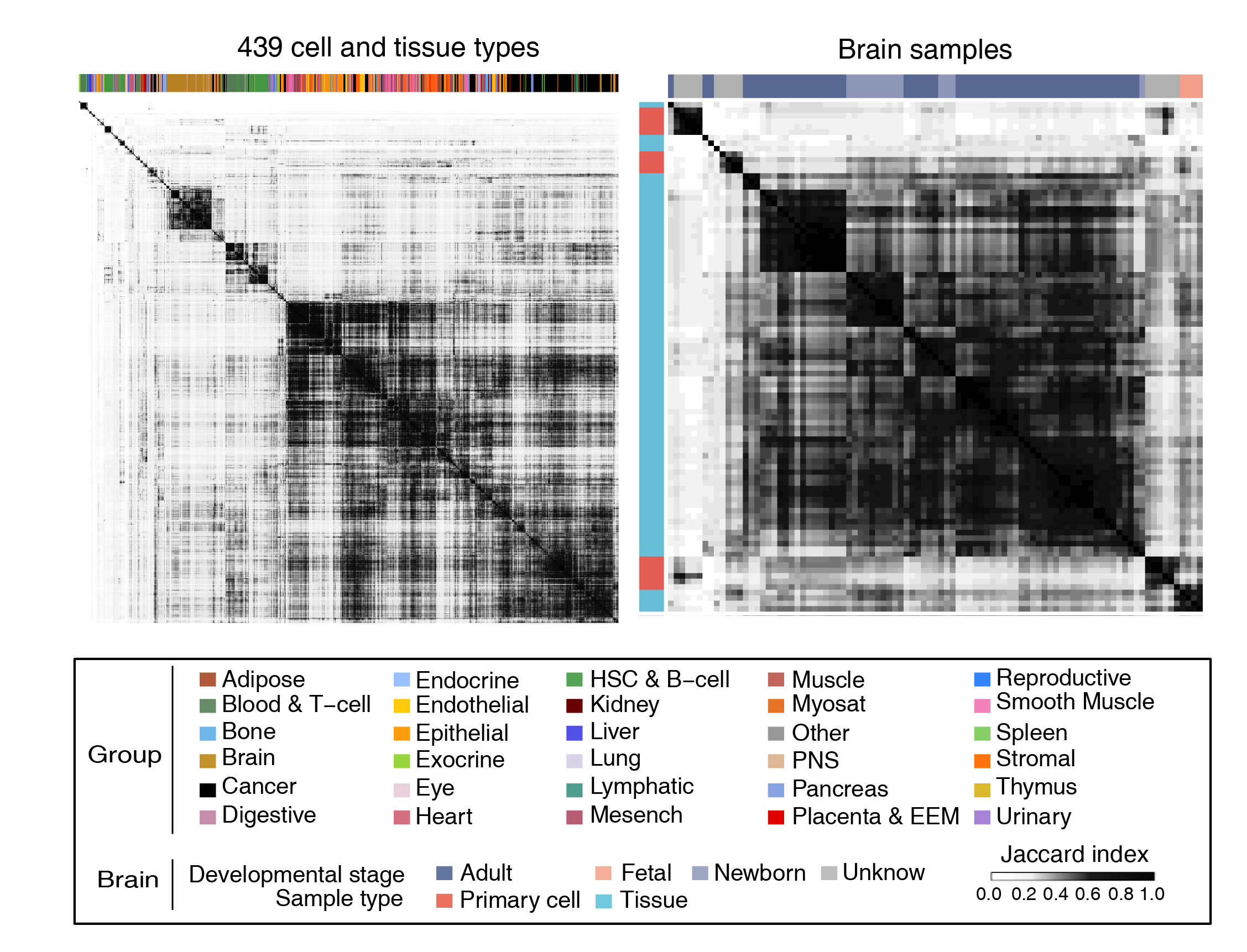


**Figure S10: Clustering of 439 tissue types and brain samples based on the similarities of their promoter activity.** We used Jaccard index to quantify the correlation of the active gene between each pair of cell and tissue types.


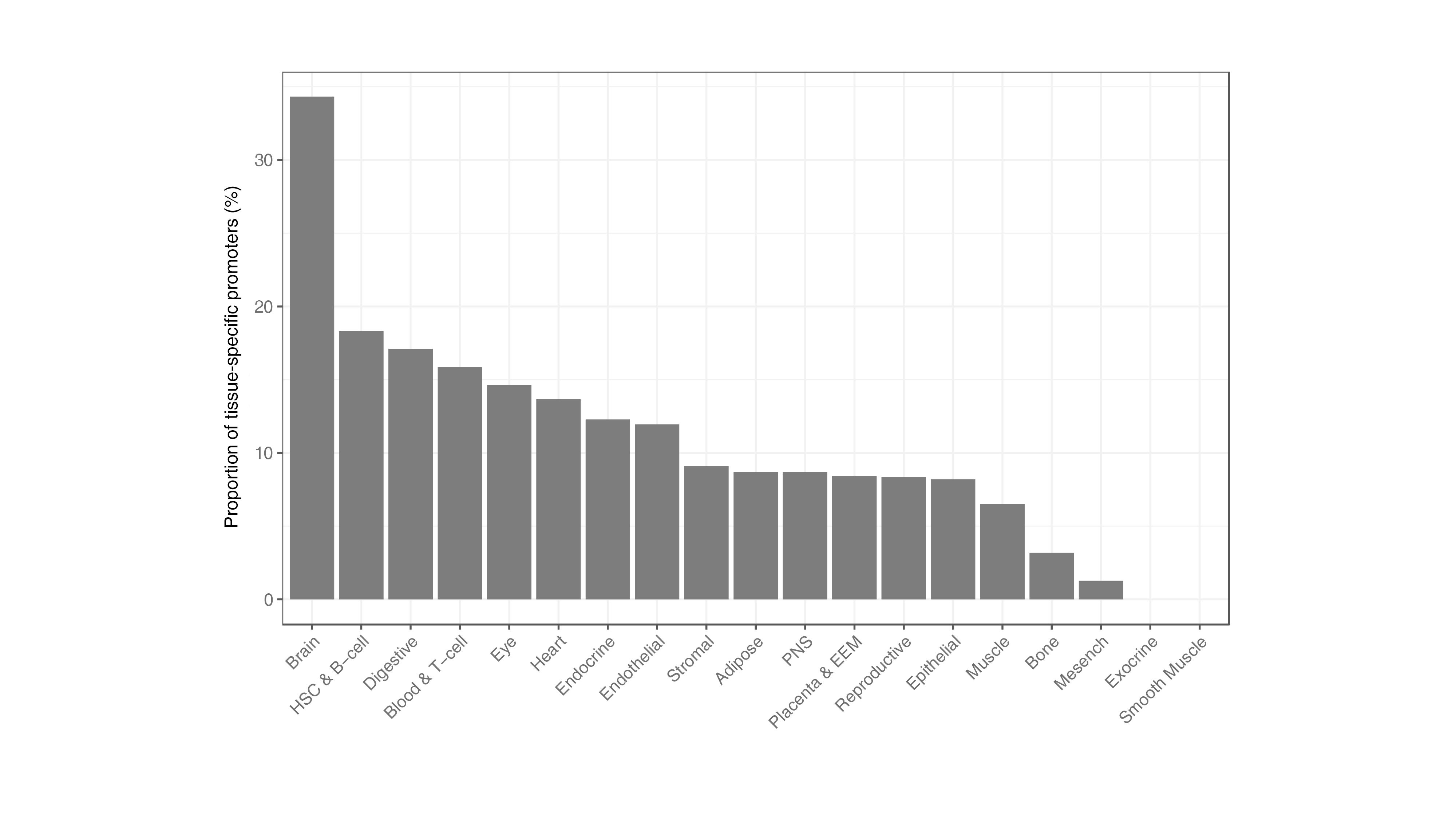


**Figure. S11: Proportion of tissue-specific promoters that are linked to tissue-specific enhancers.**


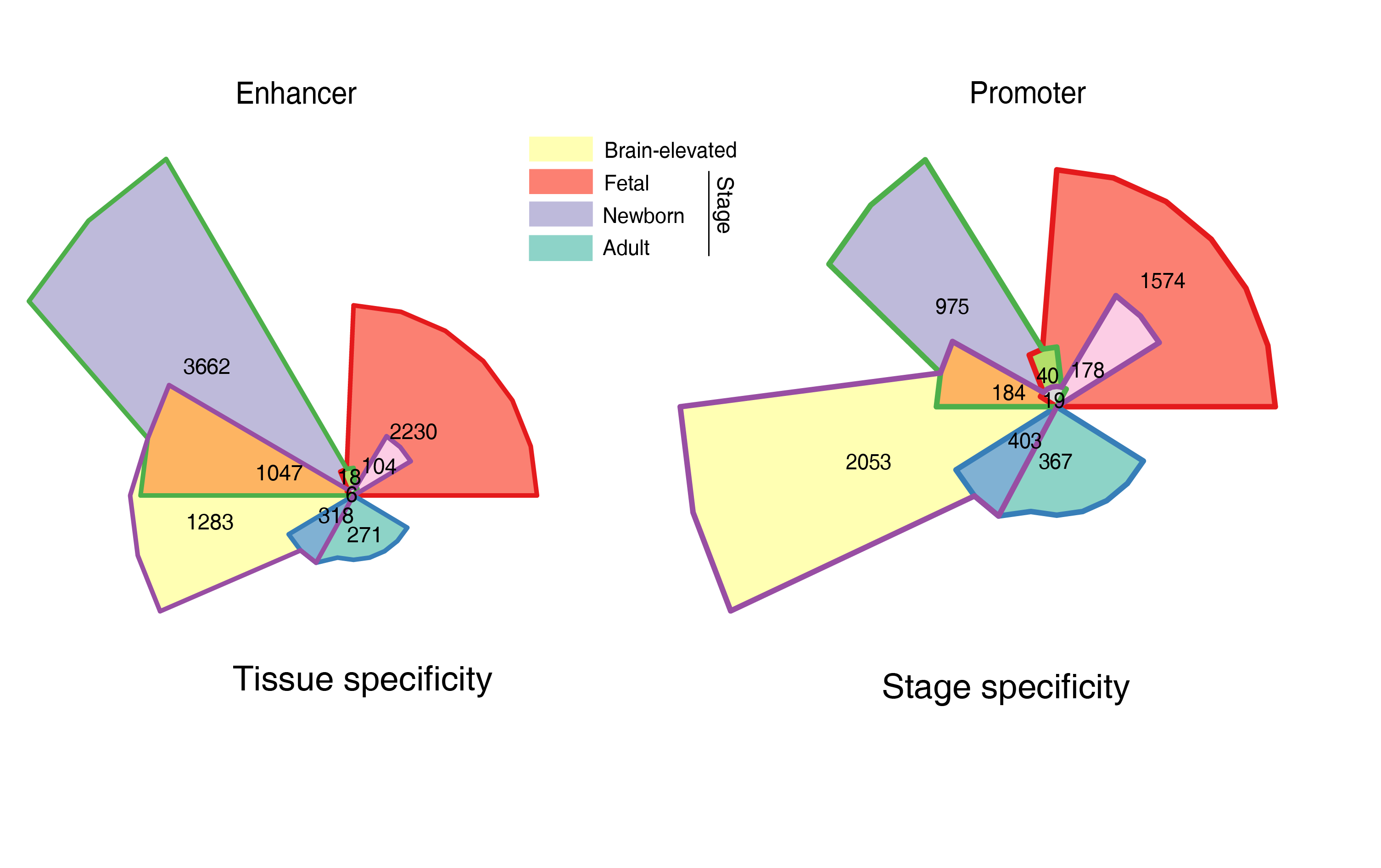


**Figure S12: Overlap between groups of enhancers and promoters with distinct tissue and stage specificity in human brain.** Note that only the enhancers and promoters retained in the reconstructed EPIs were analyzed. The enhancers and promoters in human brain were separated into different groups based on their relative CAGE activity levels compared to non-brain tissues as well as across different developmental stages.


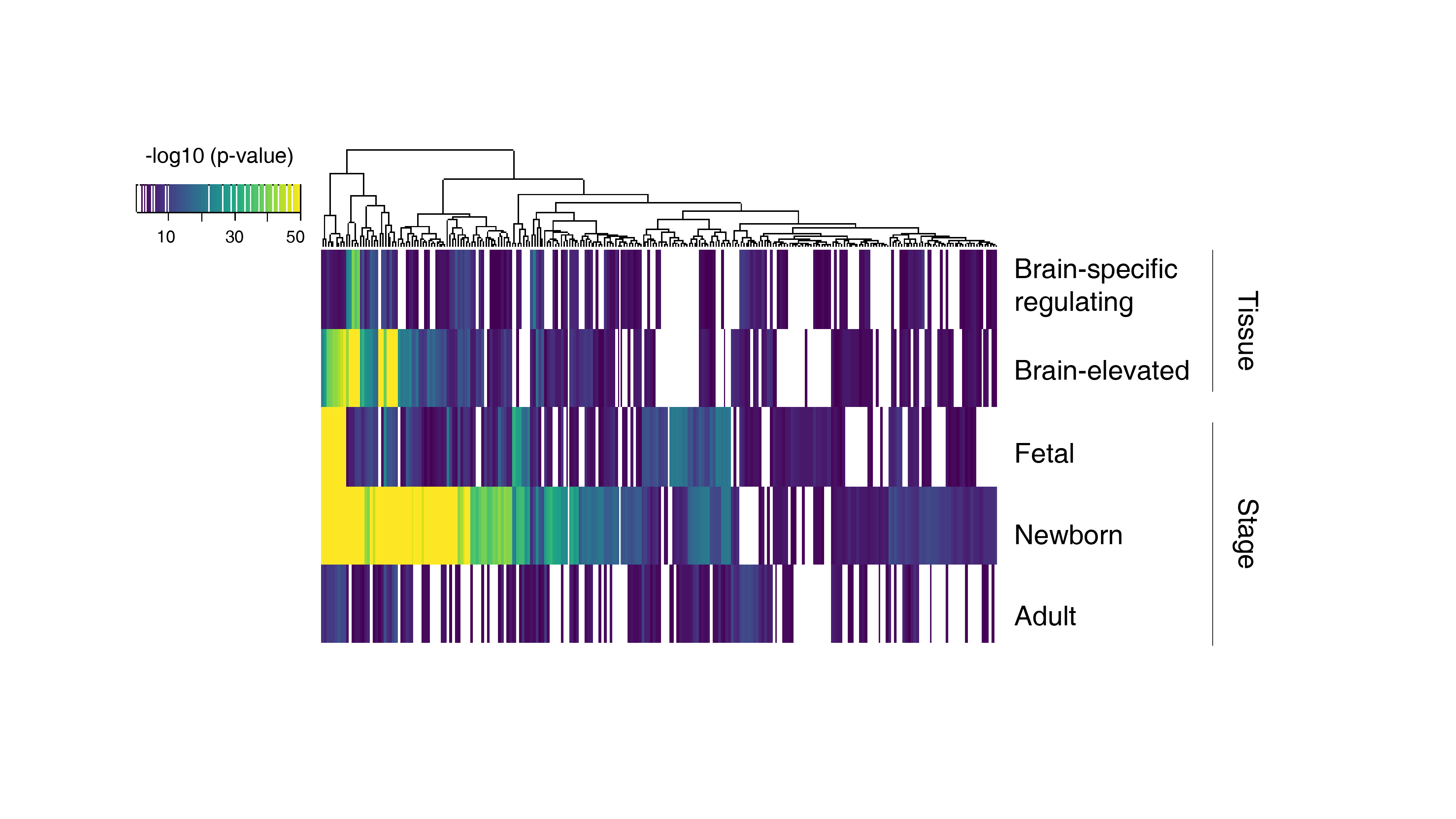


**Figure S13: The enrichment of TF binding in the enhancers with distinct tissue- (up) and stage-specific activity patterns (bottom).** In total, there are 249 distinct TFs showed binding enrichment in at least one group. Overall, TFs have stronger enrichment in the newborn than the fetal and adult brain.


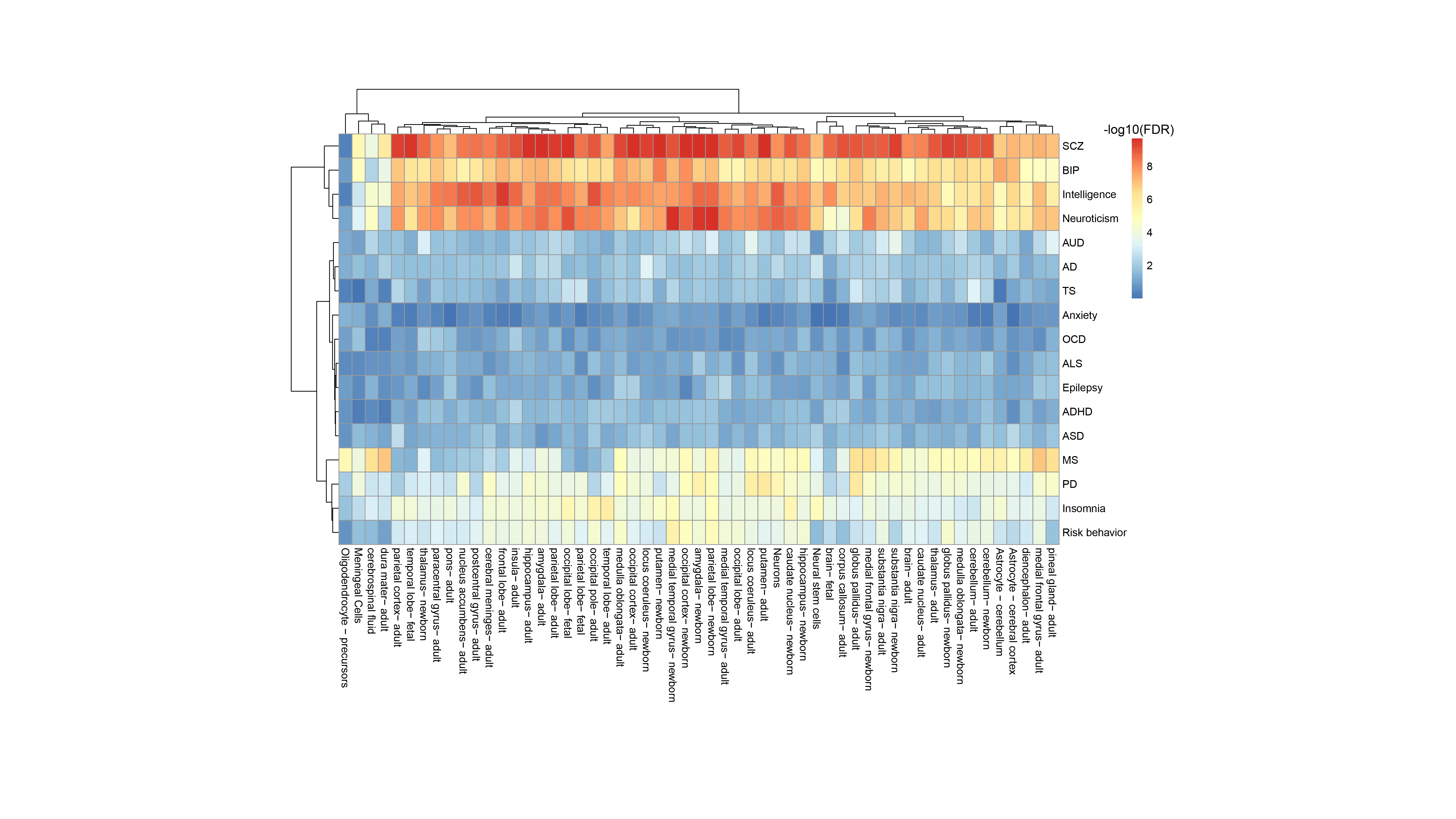


**Figure S14: Heatmap showing the partitioned heritability enrichment of genetic variants overlapping CREs from brain regions across different brain disorders and behavioral-cognitive phenotypes.** AD, Alzheimer’s Disease; ADHD, Attention Deficit Hyperactivity Disorder; ALS, Amyotrophic Lateral Sclerosis; ASD, Autism Spectrum Disorder; AUD, Alcohol Use Disorder; BIP, Bipolar Disorder; MS, Multiple Sclerosis; OCD, Obsessive-compulsive Disorder; PD, Parkinson’s Disease; SCZ, Schizophrenia; TS, Tourette's syndrome.


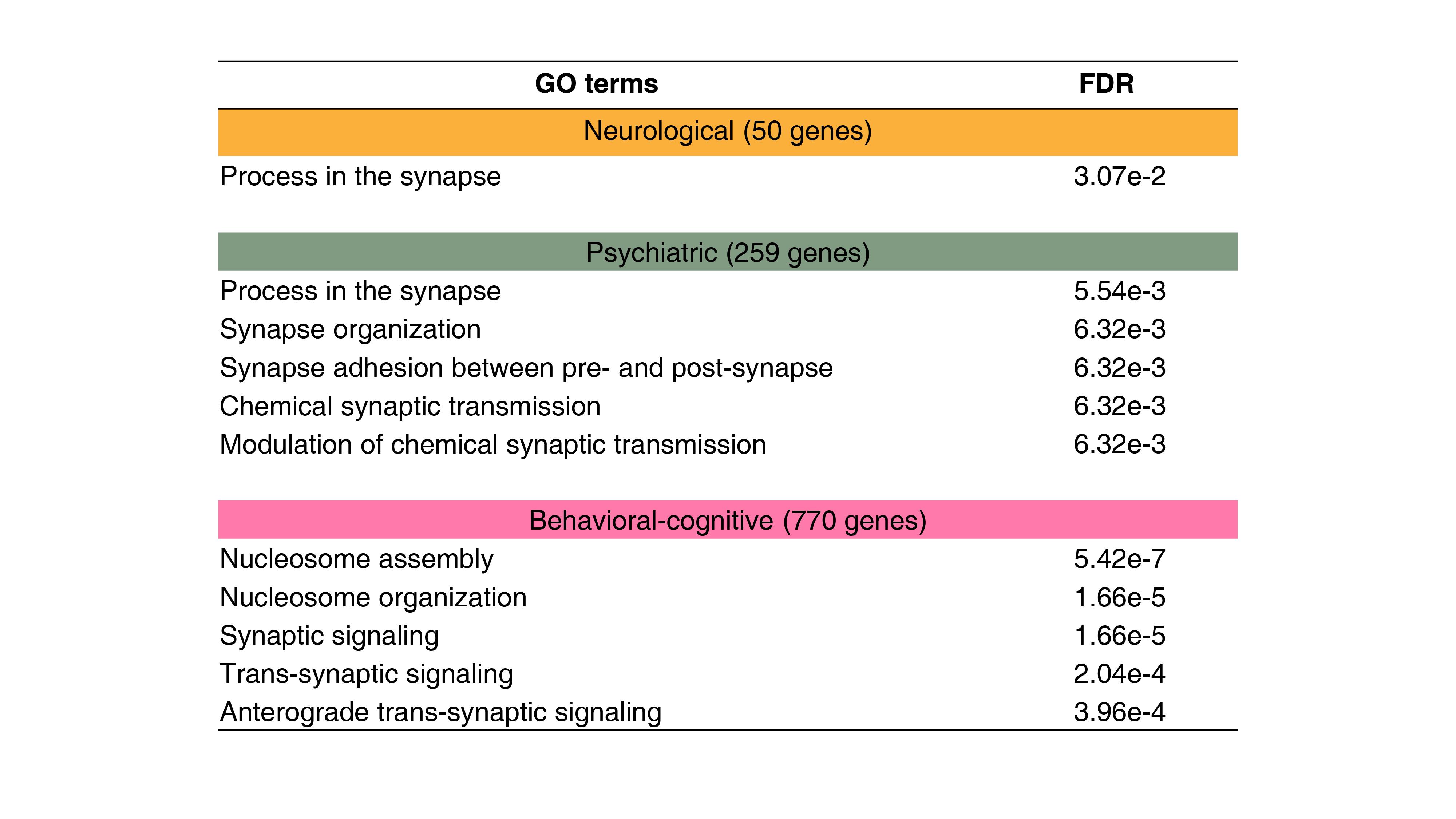


**Figure S15: The most enriched synapse-associated functions of the shared associated genes for each category of brain disorders and behavioral-cognitive phenotypes.**
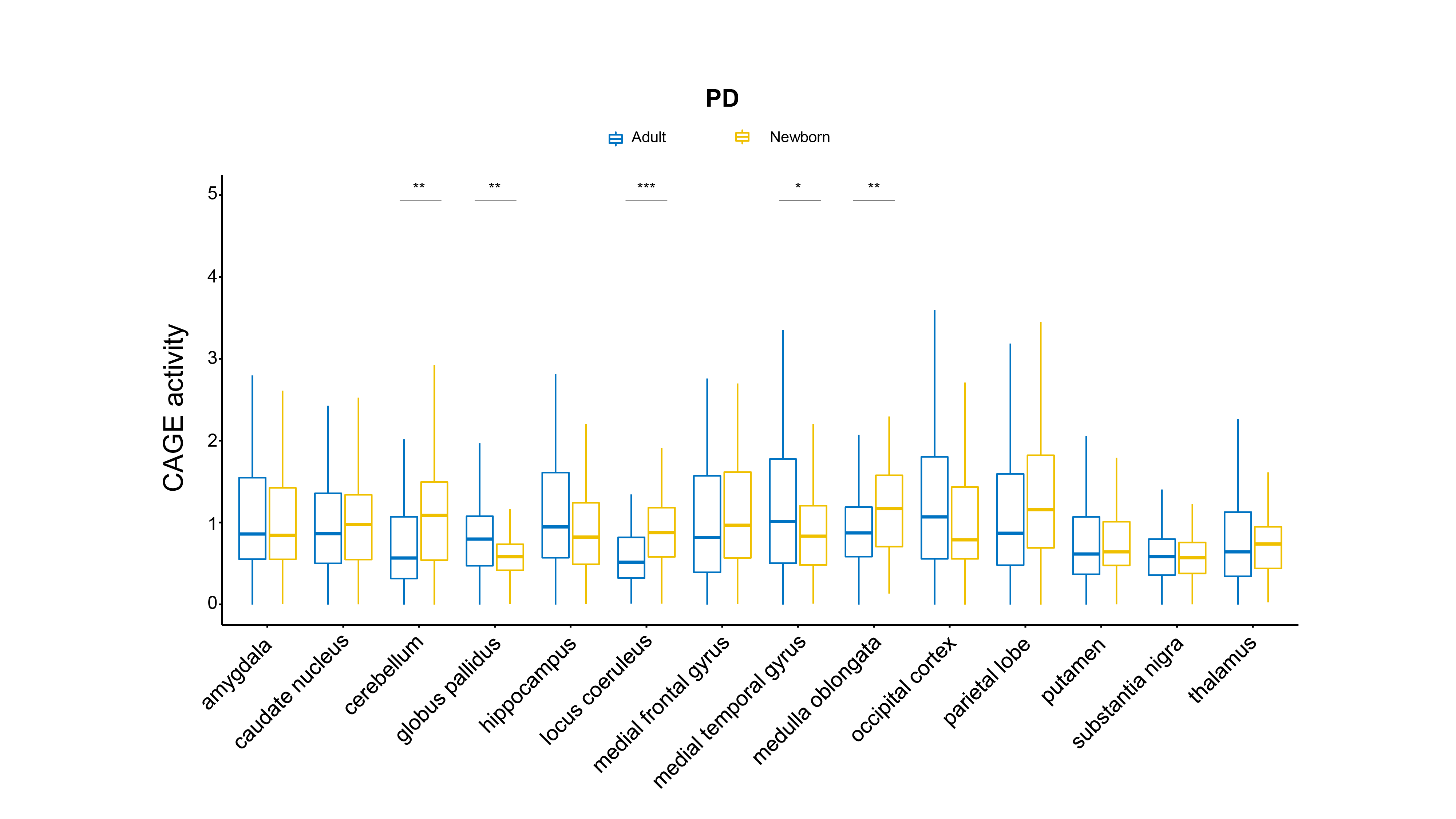


**Figure S16: CAGE activity profiles of the promoters associated with PD in diverse brain regions.** For each brain region, we showed the CAGE activity levels of the associated promoters in the newborn and adult brain, respectively. *** *P* < 0.001; ** *P* < 0.01; * *P* < 0.05; Wilcoxon test. PD, Parkinson’s Disease.


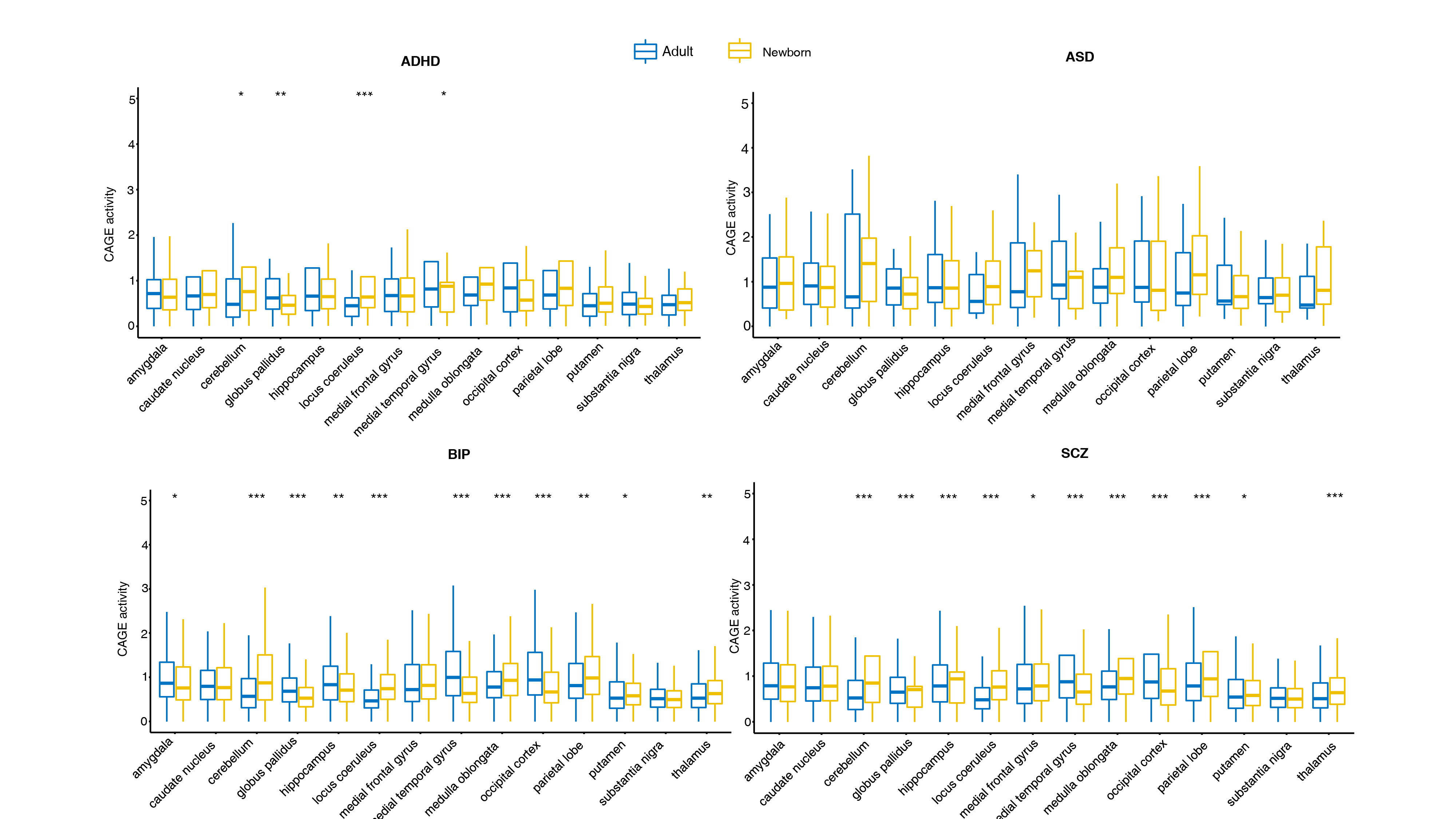


**Figure S17: CAGE activity profiles of the promoters associated with psychiatric disorders in diverse brain regions.** For each brain region, we showed the CAGE activity levels of the associated promoters in the newborn and adult brain, respectively. *** *P* < 0.001; ** *P* < 0.01; * *P* < 0.05; Wilcoxon test. ADHD, Attention Deficit Hyperactivity Disorder; ASD, Autism Spectrum Disorder; BIP, Bipolar Disorder; SCZ, Schizophrenia.


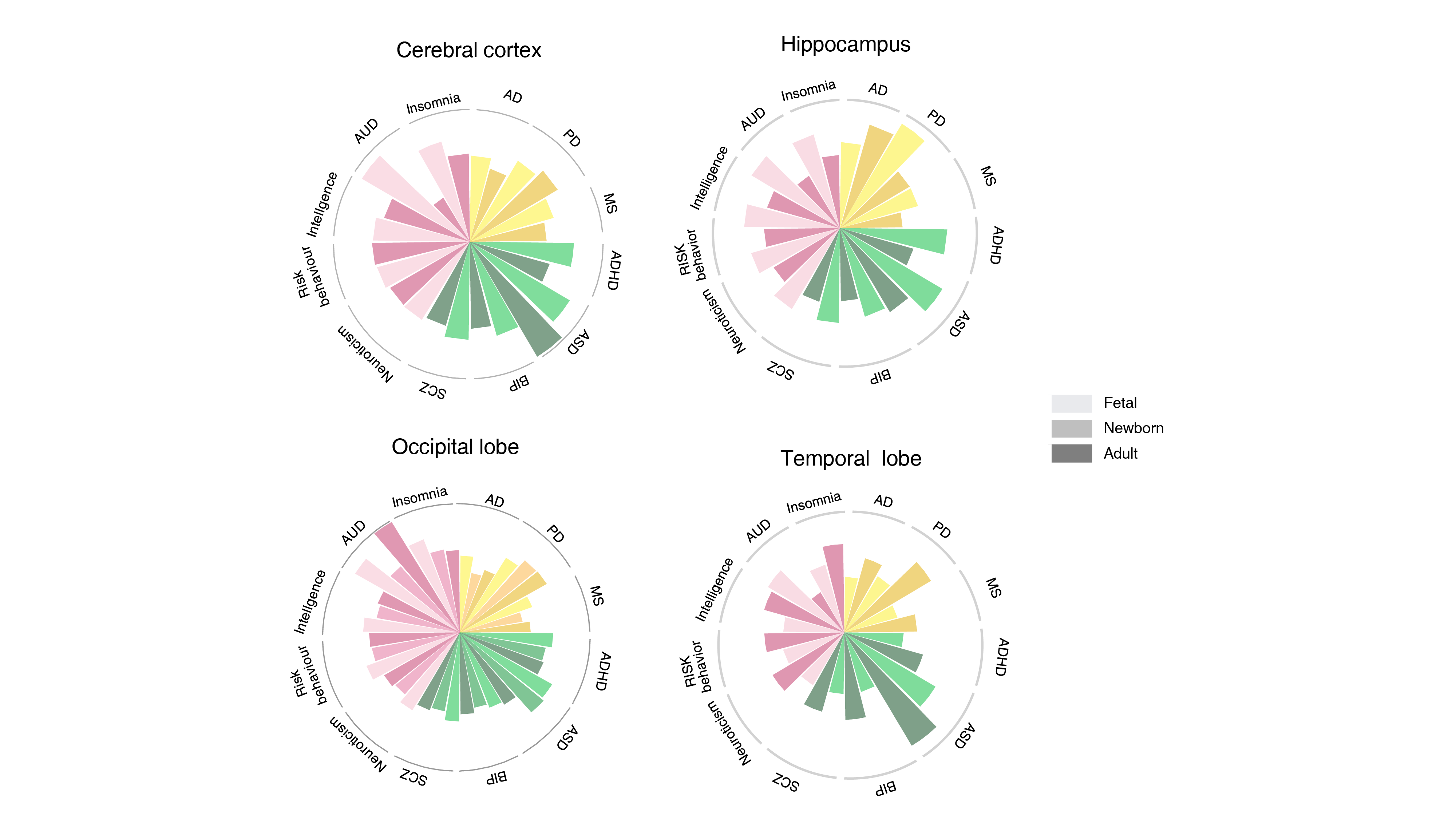


**Figure S18: CAGE activity profiles of the associated genes of diverse brain disorders and behavioral-cognitive phenotypes in specific brain regions.** For each brain disorder or behavioral-cognitive phenotype, we showed the normalized CAGE activity levels of the associated promoters in different developmental stages.


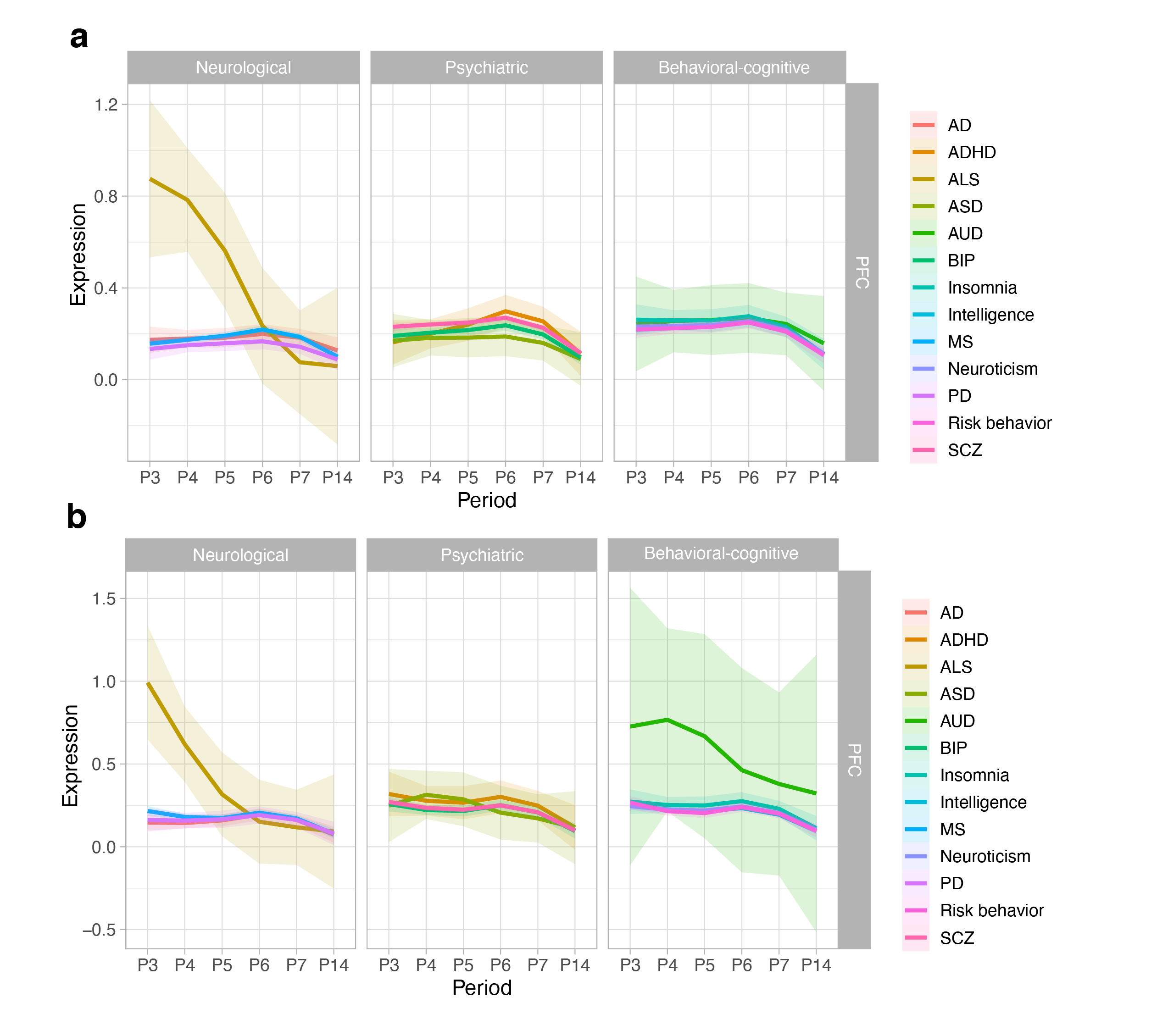


**Figure S19: Developmental expression trajectories of the associated genes in neural cell types.** We showed the single-cell expression levels of the associated genes in astrocytes (**a**) and oligodendrocyte progenitor cells (**b**). The single-cell gene expression data were collected from our STAB database [6]. P3, 10 ≤ Age < 13 PCW; P4, 13 ≤ Age < 16 PCW; P5, 16 ≤ Age < 19 PCW; P6, 19 ≤ Age < 24 PCW; P7, 24 ≤ Age < 38 PCW; P14, 40 ≤ Age < 60 Years. PCW, postconceptional weeks.

**References**

1. Boix CA, James BT, Park YP, Meuleman W, Kellis M. Regulatory genomic circuitry of human disease loci by integrative epigenomics. Nature. 2021;590:300–7. URL: https://epigenome.wustl.edu/epimap/. Accessed April, 2023.
2. Zhang K, Hocker JD, Miller M, Hou X, Chiou J, Poirion OB, et al. A single-cell atlas of chromatin accessibility in the human genome. Cell. 2021;184:5985-6001.e19. URL: http://catlas.org/humanenhancer. Accessed April, 2023.
3. Markenscoff-Papadimitriou E, Whalen S, Przytycki P, Thomas R, Binyameen F, Nowakowski TJ, et al. A chromatin accessibility atlas of the developing human telencephalon. Cell. 2020;182:754-769.e18.
4. Fulco CP, Nasser J, Jones TR, Munson G, Bergman DT, Subramanian V, et al. Activity-by-contact model of enhancer-promoter regulation from thousands of CRISPR perturbations. Nat Genet. 2019;51:1664–9.
5. GTEx Consortium. The GTEx Consortium atlas of genetic regulatory effects across human tissues. Science. 2020;369:1318–30. URL: https://gtexportal.org/home/. Accessed April 2023.
6. Song L, Pan S, Zhang Z, Jia L, Chen W-H, Zhao X-M. STAB: a spatio-temporal cell atlas of the human brain. Nucleic Acids Res. 2021;49:D1029–37. URL: https://mai.fudan.edu.cn/stab/. Accessed July 2022.
